# Time-Resolved Fluorescence Anisotropy and Molecular Dynamics Analysis of a Novel GFP Homo-FRET Dimer

**DOI:** 10.1016/j.bpj.2020.11.2275

**Published:** 2020-12-18

**Authors:** Yurema Teijeiro-Gonzalez, Alessandro Crnjar, Andrew J. Beavil, Rebecca L. Beavil, Jakub Nedbal, Alix Le Marois, Carla Molteni, Klaus Suhling

**Affiliations:** 1 Department of Physics, King’s College London, London, United Kingdom; 2 Randall Centre for Cell and Molecular Biophysics, King’s College London, London, United Kingdom; 3 Department of Cell Biology, The Francis Crick Institute, London, United Kingdom

## Abstract

Förster resonance energy transfer (FRET) is a powerful tool to investigate the interaction between proteins in living cells. Fluorescence proteins, such as the green fluorescent protein (GFP) and its derivatives, are coexpressed in cells linked to proteins of interest. Time-resolved fluorescence anisotropy is a popular tool to study homo-FRET of fluorescent proteins as an indicator of dimerization, in which its signature consists of a very short component at the beginning of the anisotropy decay. In this work, we present an approach to study GFP homo-FRET via a combination of time-resolved fluorescence anisotropy, the stretched exponential decay model, and molecular dynamics simulations. We characterize a new, to our knowledge, FRET standard formed by two enhanced GFPs (eGFPs) and a flexible linker of 15 aminoacids (eGFP15eGFP) with this protocol, which is validated by using an eGFP monomer as a reference. An excellent agreement is found between the FRET efficiency calculated from the fit of the eGFP15eGFP fluorescence anisotropy decays with a stretched exponential decay model ($\langle E_{FRET}^{exp}\rangle$ = 0.25 ± 0.05) and those calculated from the molecular dynamics simulations ($\langle E_{FRET}^{MD}\rangle$ = 0.18 ± 0.14). The relative dipole orientation between the GFPs is best described by the orientation factors $\langle \kappa^{2}\rangle$ = 0.17 ± 0.16 and 〈|κ|〉 = 0.35 ± 0.20, contextualized within a static framework in which the linker hinders the free rotation of the fluorophores and excludes certain configurations. The combination of time- and polarization-resolved fluorescence spectroscopy with molecular dynamics simulations is shown to be a powerful tool for the study and interpretation of homo-FRET.

## Significance

A new, to our knowledge, Förster resonance energy transfer (FRET) standard based on a green fluorescent protein (GFP) dimer is described, useful for reference when investigating homo-FRET in cells, e.g., when studying protein dimerization or when using homo-FRET-based biosensors. Because FRET depends on the donor and acceptor fluorophore separation and orientation, its heterogeneity for the GFP dimer may yield a multiexponential time-resolved fluorescence anisotropy decay. For this reason, we explore the stretched exponential decay model to interpret time-resolved fluorescence anisotropy decay data. With it and the support of molecular dynamics simulations, we are able to calculate the distribution of orientation factor *κ* (and *κ*^2^) and the range of distances of the two GFP fluorophores, crucial for accurately processing the experimental data.

## Introduction

The green fluorescent protein (GFP) was first extracted and purified from the jellyfish *Aequorea victoria*, whose discovery and development led to the Chemistry Nobel Prize in 2008 (1). X-ray crystallography studies revealed that GFP (27 kDa, made of 238 aminoacids) is a barrel-shaped protein, with a length of 4.2 nm and diameter of 2.4 nm (2,3). The fluorophore of the protein lies at the center of the structure, where four amino acids are responsible for the fluorescence emission. The complex photophysics of this protein and its variants has been widely studied, and protonated and deprotonated absorption bands have been identified (4, 5, 6, 7, 8). Nowadays, this protein and its genetically encoded variants are extensively used in many biological applications, e.g., fluorescence microscopy to image cells; monitoring gene expression; acting as sensors for calcium, copper, or other ions; and locating proteins, studying their interactions, and describing their dynamics (9,10).

The fluorescence decay of GFP is sensitive to the refractive index of its environment (11), which can be exploited in fluorescence lifetime imaging to map environmental changes associated with the refractive index such as protein concentration (12,13). The combination of GFP and its spectral variants in donor and acceptor pairs for Förster resonance energy transfer (FRET) allows the detection of protein interaction (14) via the donor’s fluorescence decay. In addition, biosensors have been designed according to this principle; for example, in the calmodulin calcium sensor cameleon, Ca^2+^ ions bind to the structure and induce a conformational change that is identified via FRET between a cyan and yellow fluorescent protein (15,16).

Polarization-resolved fluorescence lifetime measurements allow time-resolved fluorescence anisotropy studies. In fluorescence anisotropy studies, the rotational mobility of the fluorophore is described by the rotational correlation time, which accounts for the time it takes the fluorophore to rotate by 1 radian (17). This property is sensitive to viscosity (18), which can be imaged via time-resolved fluorescence anisotropy imaging (19).

Because of its small Stokes shift and significant overlap of absorption and emission spectra, GFP is an ideal candidate for homo-FRET, an energy transfer phenomenon in which donor and acceptor are identical. The GFP Förster distance *R*_0_, i.e., the distance at which the FRET efficiency is 50%, was reported as 4.65 ± 0.09 nm (20). Moreover, because of GFP’s large volume, its Brownian rotational diffusion is slow compared to its excited state lifetime, and it is clearly distinguishable from fast FRET. In addition, a GFP fluorescence quantum yield of 0.6 and peak extinction coefficient of 55,900 M^−1^ cm^−1^ (1,10) make GFP and its derivatives highly suitable for homo-FRET studies—much more so than small organic dyes because their fast rotational Brownian motion in fluid environments obscures homo-FRET. When FRET occurs among identical proteins in homo-FRET pairs, the transfer of nonradiative energy of one protein to the other is a reversible process. The transfer rate constants in both directions are identical if the fluorophores are in the same environment. This leads to no change in the overall emission spectrum and fluorescence lifetime (17), which is the reason why fluorescence lifetime imaging cannot be employed to study homo-FRET for GFP. Conversely, time-resolved fluorescence anisotropy can detect FRET between identical fluorescent proteins. In fact, this technique has been used in living cells to study the effect of protein dimerization and aggregation on the cell functioning (19,21, 22, 23). Although oligomerization in the biological milieu may involve a mixture of monomers and dimers, as well as higher-order oligomers, at various distances and orientations, and does not typically involve a covalent linker, the short FRET component in the anisotropy decay is a qualitative tell-tale sign of the occurrence of oligomerization. The advantage of this approach is that it can be established with a single type of fluorescence label, emitting in a single well-defined spectral region, and no two-color labeling for hetero-FRET is needed.

Several hetero-FRET constructs—in which donor and acceptor are not identical—have been described and established as FRET standards (24,25). Unlike homo-FRET, hetero-FRET is an irreversible transfer of excited state energy from the donor to the acceptor. Because it represents a de-excitation pathway for the donor, it shortens the donor decay, and it can thus be studied by observing the fluorescence decay of the donor. This is best done via time-correlated single photon counting (TCSPC) because this approach provides the lowest experimental uncertainty in the FRET efficiency (26). For example, Matthews et al. (24) investigated the fluorescence lifetime of dimers of enhanced GFP (eGFP) and the monomeric red fluorescent protein 1 (mRFP1) with different linker lengths, with eGFP and mRFP1 as donor and acceptor, respectively. They proved that when the linker length was shorter, FRET between proteins increased, yielding a decrease of the donor fluorescence lifetime and acceptor anisotropy (24). Likewise, in the work by Koushik et al., Cerulean, Venus, and Venus_*Y*67*C*_ constructs were investigated using fluorescence lifetime measurements, sensitized acceptor emission and spectral imaging: their results presented an excellent agreement (25). The unique characterization of these constructs enabled their introduction as FRET standards.

To establish FRET standards for homo-FRET studies, parameters other than the fluorescence lifetime must be employed. One example is the combination of fluorescence polarization and fluctuation analysis developed in Vogel’s group to study homo-FRET in Venus FRET constructs (27).

Single and double exponential decay models are extensively used for the interpretation of the time-resolved fluorescence anisotropy decays in the presence of FRET (28, 29, 30). However, because of its apparently more complicated form, very little has been reported on the application of the stretched exponential decay model as an FRET indicator (31, 32, 33). Here, we present a new, to our knowledge, anisotropy FRET standard formed by two eGFPs tethered by a linker of 15 amino acids (eGFP15eGFP), and describe a new protocol based on the combination of the stretched exponential decay model and molecular dynamics (MD) simulations as a tool to study homo-FRET.

## Materials and Methods

### Sample preparation

eGFP is a mutant of the wild-type GFP, with mutations of serine to threonine at position 65 (S65T) and phenylalanine to leucine at position 64 (P64L). DNA for double eGFP with a (GGGGS)_3_ linker, where G refers to glycine and S to serine, was synthesized as a double-stranded DNA gBlock (Integrated DNA Technologies, Coralville, IA), and cloned into pET151 according to manufacturer’s instructions (Thermo Fisher Scientific, Waltham, MA). eGFP alone was also cloned into pET151. This vector adds an N-terminal 6xHis and V5 tag, and a TEV cleavage site, giving an extra 33 amino acids on the N-terminus of the protein. The constructs and mutations were verified by sequencing (Eurofins Genomics, Luxembourg, Luxembourg). Both eGFP monomer and the eGFP dimer construct were provided by the Protein Production Facility of King’s College London. For expression, the constructs were transformed into BL21 Star (DE3) *Escherichia coli* (Thermo Fisher Scientific). Colonies were used to inoculate a starter culture in Luria broth containing 100 *μ*g/mL ampicillin and left shaking at 37°C for 5 h. This was used to inoculate 100 mL ZYP-5052 autoinduction media (34), and the culture was grown at 18°C shaking for 65 h. The bacteria were harvested by centrifugation at 4000 × *g* and frozen at −80°C. Pellets were thawed and EDTA-free Complete protease inhibitor tablets (Roche, Basel, Switzerland) added, and then the *E. coli* cells were lysed using BugBuster (Merck Millipore, Burlington, MA) according to the manufacturer’s instructions. The protein was found in the soluble fraction and was purified by passing over a 1 mL Histrap ff crude column (GE Healthcare, Chicago, IL) using a Bio-Rad NGC system (Hercules, CA). The column was washed with 30 column volumes of 10 mM sodium phosphate, 500 mM NaCl, 20 mM imidazole (pH 7.4) and the bound protein eluted with 10 mM sodium phosphate, 500 mM NaCl, 500 mM imidazole (pH 7.4). Fractions containing proteins were then pooled and concentrated (Amicon Ultra 15; Merck Millipore), then further purified using size exclusion chromatography. Using a Gilson HPLC system (Madison, WI), samples were run on a Superdex 200 increase 10/300 GL column in phosphate-buffered saline (PBS) at pH 7.4 (OXOID). Fractions corresponding to the monomer for each construct were pooled and concentrated as required, resulting in different amounts of stock solution for GFP monomer and GFP dimer. For the measurements, the concentrations [*C*] for monomer and dimer were [*C*]_*monomer*_ = 0.89 *μ*M and [*C*]_*dimer*_ = 0.34 *μ*M, respectively, in 0%, 5%, 10%, 15%, 20%, 25%, and 30% and 35%, 45%, and 50% (v/v) glycerol in PBS solutions. This corresponds to an average nearest neighbor distance *d* = 68 nm between the GFP monomers and *d* = 94 nm between the dimers, with *d* = 0.55/[*C*]^1/3^, with [*C*] quoted in fluorophores per volume (35,36). With an average decay time *τ* of 2.6 ns, the diffusion length *l*, i.e., the average distance traveled in the excited state, given by *l* = $\sqrt{6D\tau }$, with *D* the diffusion coefficient (*D*_*GFP*_ = 0.87 × 10^−10^ m^2^/s (37)), is l = 1.2 nm. This is much shorter than the nearest-neighbor distance, and interaction between the individual eGFP monomers or eGFP dimers is thus insignificant at the concentrations used in this work.

### Steady-state spectra

Steady-state polarization-resolved excitation and emission spectra were obtained on a luminescence spectrometer (LS-5; Perkin-Elmer, Waltham, MA) using a 1 cm pathlength quartz cuvette. Four measurements were taken per experiment using two polarizers, one located between the excitation source and the sample and the second between the sample and the emission detector: *I*_*VV*_(*λ*), *I*_*VH*_(*λ*), *I*_*HH*_(*λ*), and *I*_*HV*_(*λ*), where subscript *V* refers to the vertical and *H* to the horizontal polarization for excitation and emission, respectively. Measurements were taken from *λ*_*exc*_ = 350 nm to *λ*_*exc*_ = 520 nm in 2 nm steps. Fluorescence emission was detected at 530 nm. The data analysis was carried out as described previously, taking into account the spectral sensitivity of the spectrometer (38). The emission spectrum was recorded on the same setup without polarizers. The excitation wavelength was *λ*_*exc*_ = 450 nm. Fluorescence emission was detected from *λ* = 460 nm to *λ* = 660 nm every 2 nm.

The absorption spectrum was obtained by using an absorption spectrometer (U-4100 dual-beam spectrometer; Hitachi, Tokyo, Japan), with a 1 cm pathlength quartz cuvette. The absorption spectrum was recorded from 330 nm to 560 nm in 2 nm steps, and the solvent (buffer) absorption spectrum was subtracted. The spectral overlap *J*(*λ*) between the absorption and emission spectra was calculated via the a|e UV-Vis-IR Spectral Software (39), where the GFP peak extinction coefficient at 488 nm, *ɛ*_488_ = 55,900 M^−1^ cm^−1^, was introduced as an input (10).

### Time-resolved fluorescence anisotropy measurements

**Figure 1:**
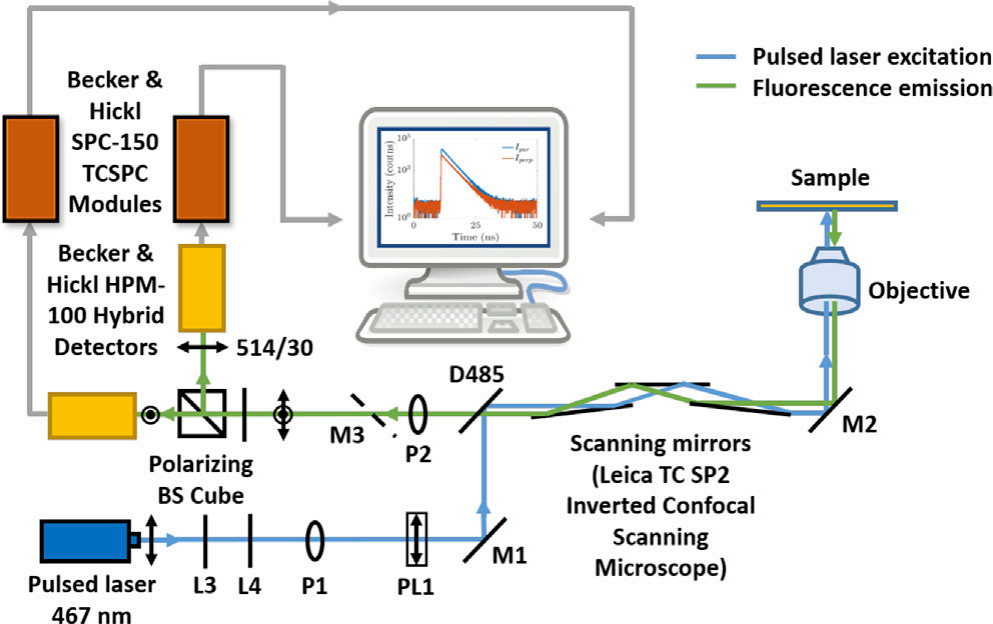
Schematic of the experimental setup. M stands for mirror, L for lens, P for pinhole, D for dichroic, and PL for polarizer. To see this figure in color, go online.

Single fluorescence decays were measured on an inverted confocal microscope (TCS SP2; Leica, Wetzlar, Germany). Excitation was provided by a 467 nm diode laser (PLP-10 470; Hamamatsu, Hamamatsu, Japan) at a repetition rate of 20 MHz with an average power in the microwatt region. Time-resolved fluorescence anisotropy experiments were performed with two TCSPC cards (SPC-150; Becker & Hickl, Germany) connected to two hybrid detectors (HPM-100-40; Becker & Hickl). Fluorescence emission, passed through a 514/30 bandpass filter, was separated into two orthogonal polarization components by a polarizing beam splitter cube (Edmund Optics, Barrington, NJ) before reaching the hybrid detectors. The acquisition time was 5 min, and the TCSPC time resolution was 4096 bins of 12 ps each. We calculated the fluorescence anisotropy decays and also the fluorescence decays from these measurements. In addition, the fluorescence lifetime measurements were performed without the polarizing beam splitter cube, using a single detector and one TCSPC card only for solutions up to 30% glycerol. A sample volume of 250 *μ*L was measured in an eight-well coverslip bottom plate (ibidi, Gräfelfing, Germany) at room temperature. The schematic of the setup is presented in Fig. 1.

### Refractive index measurements

The refractive index was measured using an Abbe refractometer at room temperature. A tungsten lamp (visible range) was used, and the system was calibrated with water, whose value is very well known (n = 1.336 at *λ* = 589 nm and T = 20°C) (40). Three readings were averaged per measurement.

### MD simulations

The model of the GFP monomer was created from an x-ray structure solved at 0.19 nm resolution (Protein Data Bank: 1GFL) (3). The internal fluorophore was taken from the eGFP Protein Data Bank, PDB: 2Y0G (41) and inserted in the homologous GFP model using the software Yasara (42). To build the GFP15GFP dimer, we duplicated the monomer structure and translated the copy so that the two monomers were separated from each other by ∼2 nm. This two-domain template was then uploaded in the modeling server SWISS-MODEL (43, 44, 45, 46, 47) to build a linker of sequence GGGGSGGGGSGGGGS. The resulting structure of this linker was characterized by a certain degree of folding because of the distance between the two monomers. Both the monomer and the dimer were solvated with a 1.2 nm water buffer in a truncated octahedral periodically repeated supercell. Na^+^ ions were added to neutralize the charge of the system because each monomer contains 21 negative charges. Overall, the monomer system contains 29,922 atoms, whereas the dimer system contains 123,999 atoms. Simulations were performed with the wild-type GFP, whereas the experiments were carried out using eGFP with a cleavable HIS-tag. The structures of these types of GFP are identical; only the fluorophores are slightly different, and we do not expect the Brownian rotation of the protein to be affected by this distinction.

The AMBER ff14sb force field (48) was used for both the monomer and the dimer. The internal fluorophore was parameterized with the General AMBER force field, and its partial charges were assigned according to the AM1-BCC charge scheme (49,50). As in previous simulation studies of fluorescence anisotropy (51), it was assumed that the ground state interaction potential can also be representative of the excited states. The proteins were solvated in water, which has a similar viscosity to PBS, used in the experiment. The commonly used TIP3P (52) water model was chosen. The low value of the viscosity of this water model with respect to experiments (53,54) was taken into account by a suitable rescaling procedure when calculating rotational correlation times, as described in the Results. The rotational correlation time is related to the solution viscosity via the Stokes-Einstein-Debye relationship (17):

(1) $$\theta =\frac{\eta V}{k_{B}T},$$

where *k*_*B*_ is the Boltzmann constant, *T* the absolute temperature, *η* the environmental viscosity, and *V* the volume of the fluorophore.

Molecular dynamics simulations of the GFP monomer and dimer were carried out with AMBER 12 (PMEMD) (55). The SHAKE algorithm was used to restrain bonds containing hydrogens, and a time step of 2 fs was used (except for the NPT equilibration, for which a time step of 1 fs was used), and a cutoff of 1 nm was used for the nonbonded interactions. Long-range electrostatic interactions were evaluated with Particle Mesh Ewald. The system was first minimized by restraining the protein with a harmonic potential of spring constant 10^3^ kcal mol^−1^ nm^−2^ and then without any restraint. It was then heated in the canonical (NVT) ensemble for 1 ns (0.5 ns in the case of the monomer) from 0 to 300 K by means of the weak-coupling Berendsen algorithm with a time constant of 0.5 ps (56), keeping the protein restrained. It was equilibrated at 1 bar in an isothermal-isobaric (NPT) ensemble for 5 ns (2 ns in the case of the monomer) by means of a Langevin thermostat with collision frequency of 2.0 ps^−1^ and a Berendsen barostat with time constant of 0.5 ps. Finally, a production of 500 ns in the microcanonical (NVE) ensemble was carried out. The NVE ensemble was chosen because it does not disrupt diffusion processes such as the rotational diffusion we are interested in.

The fluorescence anisotropy decay can be represented by the autocorrelation function (ACF) of the normalized transition dipole moment direction (51,57, 58, 59, 60), which is defined as a single unit vector for each fluorophore. There is one for the monomer and two for the dimer (51); in the latter case, the anisotropy is calculated as the average of the anisotropies of each of the two monomers.

**Figure 2:**
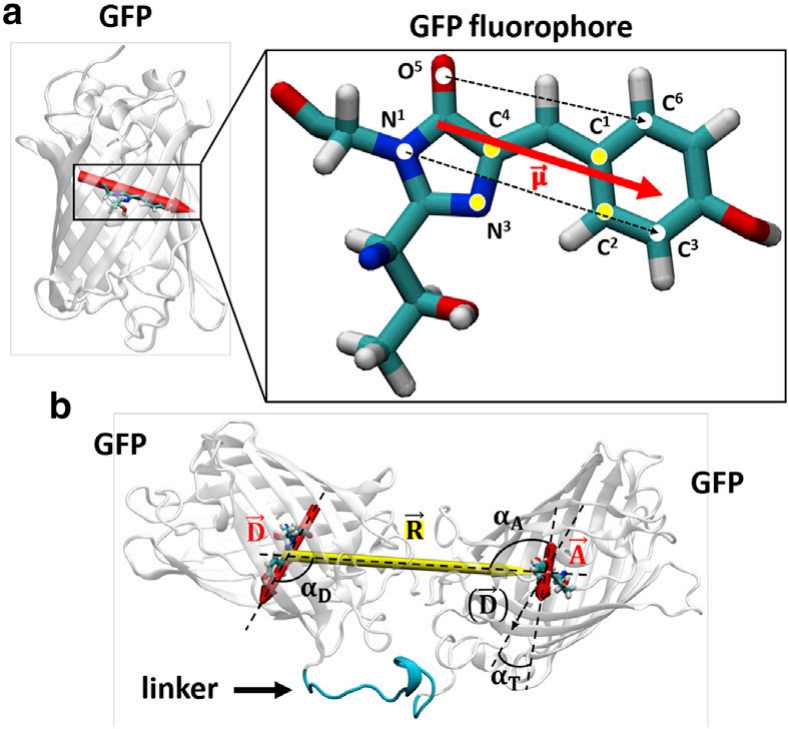
Illustration of the GFP15GFP dimer configuration and the linker. (*a*) The fluorophore responsible for the fluorescence emission is shown in the enlarged image, in which the atoms used for defining the transition dipole moment and the distance between proteins are indicated. The transition dipole moment $\vec{\mu }$ is represented as a red arrow, and its direction is that of the average of the two vectors that link atoms C^6^-O^5^ and C^3^-N^1^ (*dashed black arrows*). (*b*) A representative GFP15GFP configuration. Donor $\left(\vec{D}\right)$ and acceptor $\left(\vec{A}\right)$ transition dipole vectors are shown in red. The distance $\vec{R}$ between the fluorophores is shown in yellow (and the four atoms whose center of mass is used for each of the two ends of $\vec{R}$ are shown in *yellow* in the enlarged image in *a*). The angles between the vectors are *α*_*D*_ (between $\vec{D}$ and $\vec{R}$), *α*_*A*_ (between $\vec{A}$ and $\vec{R}$), and *α*_*T*_ (between $\vec{D}$ and $\vec{A}$). The flexible linker that connects the two GFPs is shown in blue. To see this figure in color, go online.

The fluorescence anisotropy decay of a rotating unit is given by the following expression:

(2) $$r\left(t\right)=\frac{2}{5}{\langle \frac{3{\left(\vec{\mu }\left(t_{0}\right)•\vec{\mu }\left(t_{0}+t\right)\right)}^{2}}{2}-\frac{1}{2}\rangle }_{t_{0}},$$

where $\vec{\mu }\left(t\right)$ is a given normalized transition dipole moment as a function of time and the brackets indicate an average over every possible initial time *t*_0_. A discrete version of Eq. 2 that can be implemented for the postproduction of an MD simulation trajectory is given by:

(3) $$r\left(t_{i}\right)\approx \frac{2}{5}\frac{1}{T_{tot}-t_{i}}\overset{T_{tot}-1-t_{i}}{\underset{t_{j}^{\text{′}}=0}{\sum }}\frac{3{\left(\vec{\mu }\left(t_{j}^{\text{′}}\right)•\vec{\mu }\left(t_{j}^{\text{′}}+t_{i}\right)\right)}^{2}-1}{2},$$

where *t*_*i*_ and *t*′_*j*_ are the *i*-th and *j*-th time steps, respectively, and *T*_*tot*_ is equal to the number of time steps in the MD trajectory. In the case of GFP, $\vec{\mu }$ can be defined as the normalized average of the vector connecting atoms C^6^ and O^5^ and the one connecting atoms C^3^ and N^1^ in the fluorophore (as shown in Fig. 2 *a*; (61,62)).

Under the assumption of ergodicity, we divided the 500-ns-long trajectories into 10 trajectories of 50 ns each. The ACF, with a time resolution of 10 ps, was then computed on the last nine and averaged, and the first was discarded for equilibration reasons. This way of dividing the trajectory was chosen to provide large enough statistics for averaging the ACF while at the same time providing a long enough time window for the average ACF to be fitted over.

From the MD trajectories, the FRET orientation factor *κ*^2^ was calculated as (61):

(4) $$\kappa^{2}={\left(\left(\vec{D}\cdot \vec{A}\right)-3\left(\vec{D}\cdot \vec{R}\right)\left(\vec{A}\cdot \vec{R}\right)\right)}^{2},$$

where $\vec{D}$ and $\vec{A}$ are the normalized transition dipole moments of the donor and acceptor fluorophores, respectively, and $\vec{R}$ is the normalized distance vector between the two fluorophores. Here, this was calculated as the average of the four vectors linking the coordinates of four atoms in the donor fluorophore (the surrogate GFP benzylidene C^1^ and C^2^ and imidazolone N^3^ and C^4^) with their corresponding ones within the acceptor fluorophore, as shown in Fig. 2 *a* (61).

Equation 4 can be rewritten as a function of the angle between vectors:

(5) $$\kappa^{2}={\left(\text{cos}\alpha_{T}-3\text{cos}\alpha_{D}\text{cos}\alpha_{A}\right)}^{2},$$

where *α*_*T*_, *α*_*D*_, and *α*_*A*_ are the angles between $\vec{D}$ and $\vec{A}$, $\vec{D}$ and $\vec{R}$, and $\vec{A}$ and $\vec{R}$, respectively.

The FRET efficiency was calculated from the relative dipole orientation between the two GFPs through *κ*^2^ and their separation *R* as (17):

(6) $$E_{FRET}=\frac{1}{{\left(\frac{R}{R_{0}}\right)}^{6}+1},$$

where *R*_0_ contains *κ*^2^ and is the so-called Förster distance at which the energy efficiency due to FRET is a half. It is given by (17):

(7) $$R_{0}=0.021{\left(\kappa^{2}n^{-4}\Phi J\left(\lambda \right)\right)}^{1/6}\left[\text{nm}\right],$$

where *Φ* is the quantum yield of the donor in the absence of the acceptor, *n* is the refractive index of the environment where the FRET takes place, and *J*(*λ*) is the overlap integral of the absorption spectrum of the acceptor and the emission spectrum of the donor.

### Data analysis

The parallel and perpendicular fluorescence intensity decays—*I*_||_(*t*) and $I_{⊥}\left(t\right)$, respectively—were used to produce the experimental time-resolved fluorescence anisotropy decay using the following equation (17):

(8) $$r\left(t\right)=\frac{I_{∥}\left(t\right)-GI_{⊥}\left(t\right)}{I_{∥}\left(t\right)+2GI_{⊥}\left(t\right)},$$

where *G* corresponds to the efficiency ratio between detection paths.

The fluorescence anisotropy decay of monomeric eGFP was fitted with a single exponential decay model:

(9) $$r\left(t\right)=r_{0}e^{-t/\theta },$$

where *r*_0_ is the initial anisotropy and *θ* is the rotational correlation time (17). The eGFP15eGFP fluorescence anisotropy decay was fitted with double (28, 29, 30) and stretched exponential (32,63) decay models. The stretched exponential decay model was given by the following expression in the static regime, in which the donor’s fluorescence decay in the presence of an acceptor is much shorter than its rotational rate:

(10) $$r\left(t\right)=r_{0}e^{-\gamma_{st}t^{\delta }},$$

where *δ* is the dimensionality of the system. If the system is three-dimensional, then *δ* = 1/2, and for two dimensions, *δ* = 1/3 (31,64).

*γ*_*st*_ is given by:

(11) $$\gamma_{st}=\sqrt{\frac{\pi }{2\tau }}\;c{\left(\frac{3}{2}\right)}^{1/2}\langle \left|\kappa \right|\rangle ,$$

where 〈|κ|〉 accounts for the mean of the absolute value of the orientation factor *κ*, *τ* is the fluorescence lifetime of the donor in the absence of any acceptor, and *c* is a dimensionless parameter that accounts for the number of fluorophores within a space of radius *R*_0_.

The FRET energy efficiency, calculated from the application of the stretched exponential decay model to the anisotropy decay, is given by (32,65,66):

(12) $$E_{FRET}=\sqrt{\pi }ye^{y^{2}}\left[1-\text{erf}\left(\text{y}\right)\right],$$

where *y* = $\frac{\sqrt{\tau }}{2}\gamma_{st}$ and *erf* is the error function.

Additional information about the derivation and relationship between parameters of the stretched exponential decay model is provided in the Supporting Materials and Methods.

An alternative model to interpret the eGFP15eGFP anisotropy decay is the double exponential function, in which one component accounts for rotational motion and one component accounts for homo-FRET:

(13) $$r\left(t\right)=r_{01}e^{-t/\theta }+r_{02}e^{-t/\phi },$$

where *θ* is the rotational correlation time, *ϕ* the inverse FRET rate constant, and *r*_01_ and *r*_02_ the initial anisotropy.

For a homo-FRET dimer, the single-step energy transfer rate *k*_*T*_ occurs in both ways identically. For this reason, the relationship between *ϕ* and *k*_*T*_ is given by *k*_*T*_ = 1/2*ϕ* (22,67). Knowing this relationship and that *k*_*T*_ depends on the sixth power of the separation between fluorophores *R* (*k*_*T*_ = *τ*^−1^(*R*_0_/*R*)^6^ (65)), Eq. 6 can be rewritten as follows:

(14) $$E_{FRET}=\frac{k_{T}}{\tau^{-1}+k_{T}},$$

where *τ* is the fluorescence lifetime of the isolated donor, i.e., the average time it takes for the excited fluorophore to return to its ground state.

To obtain the FRET efficiency using Eq. 14, the average fluorescence lifetime of the monomeric eGFP was calculated from the denominator of Eq. 8 $\left(I\left(t\right)=I_{∥}\left(t\right)+2GI_{⊥}t)\right)$ and fitted with a double exponential decay model, which was convolved with the instrument response function (IRF) (17,68):

(15) $$I\left(t\right)=IRF\times \left(A_{1}e^{-t/\tau_{1}}+A_{2}e^{-t/\tau_{2}}\right),$$

where *A*_*i*_ corresponds to the fluorescence intensity contribution of each fluorescence lifetime *τ*_*i*_.

The intensity-averaged lifetime *τ*_*avg*_ was calculated as (69):

(16) $$\tau_{avg}=\frac{A_{1}\tau_{1}^{2}+A_{2}\tau_{2}^{2}}{A_{1}\tau_{1}+A_{2}\tau_{2}}.$$

The dependence of the average eGFP and eGFP15eGFP fluorescence lifetime on its environmental refractive index was assessed using the Strickler-Berg formula (70):

(17) $$k_{r}=2.88\times 10^{-9}n^{2}\frac{\int F\left(\tilde{\nu }\right)d\tilde{\nu }}{\int F\left(\tilde{\nu }\right){\tilde{\nu }}^{-3}d\tilde{\nu }}\int \frac{\varepsilon \left(\tilde{\nu }\right)d\tilde{\nu }}{\tilde{\nu }},$$

where *k*_*r*_ is the radiative rate constant and is related to the fluorescence lifetime *τ* and the nonradiative rate constant *k*_*nr*_ by *τ* = $1/(k_{r}+k_{nr})$. *F* is the fluorescence emission, *ε* the extinction coefficient, and $\tilde{\nu }$ the wavenumber ($\tilde{\nu }=1/\lambda$, with *λ* wavelength).

Fluorescence decays were analyzed and plotted with a home-built MATLAB (The MathWorks, Natick, MA) script, in which the Levenberg-Marquardt algorithm was employed to fit the data via the nonlinear least-squares method.

The viscosity of each solution was calculated by the method developed by Nian-Sheng Cheng (71), which takes into account the water/glycerol ratio of the solution and its temperature.

Simulated fluorescence anisotropy decays calculated from an ACF were fitted with a single exponential decay model (Eq. 9) and hindered rotation decay model:

(18) $$r\left(t\right)=\left(r_{0}-r_{\infty }\right)e^{-t/\theta }+r_{\infty },$$

where *r*_∞_ accounts for a hindered rotation (17) for GFP and GFP15GFP, respectively. The ratio *r*_∞_/*r*_0_ can be used to calculate the semicone angle *θ*_*c*_ in the wobble-in-a-cone model (72,73):

(19) $$\frac{r_{\infty }}{r_{0}}=\frac{1}{2}cos\theta_{c}\left(1+cos\theta_{c}\right).$$

From the rotational correlation time, the solution viscosity was calculated using the Stokes-Debye-Einstein relationship (Eq. 1).

The trajectories postproduction were analyzed with MDAnalysis (74,75) and CPPTRAJ (76). VMD was used for visualizing the trajectories (77). All simulated data were plotted in MATLAB, and similarly to the experimental data, the fits were performed using the nonlinear least-squares method and the Levenberg-Marquardt algorithm.

## Results and Discussion

### Red-edge excitation of eGFP15eGFP confirms homo-FRET

**Figure 3:**
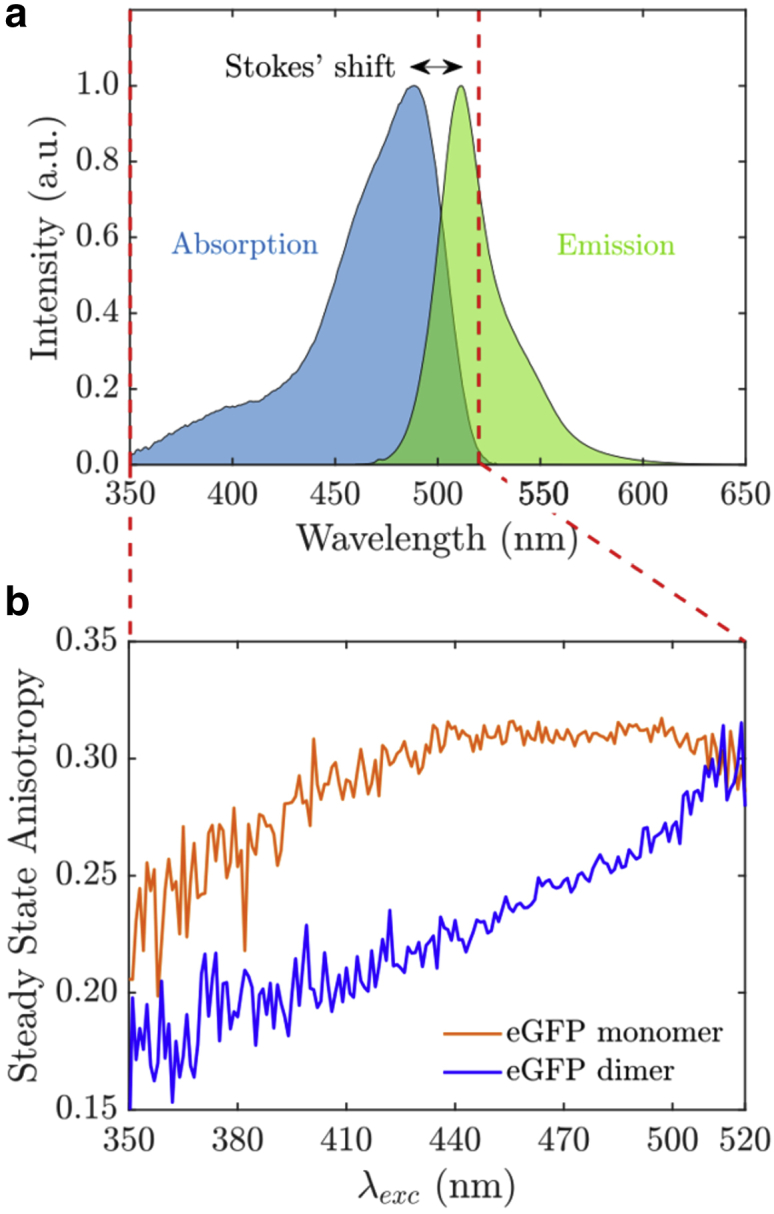
(*a*) Absorption and emission spectra of eGFP in PBS. (*b*) Steady-state anisotropy measurements at different excitation wavelengths for eGFP monomer and dimer in PBS are given. To see this figure in color, go online.

Fig. 3 *a* shows the absorption and emission spectra of eGFP in PBS. The overlap spectrum *J*(*λ*) in Eq. 7 was calculated to be *J*(*λ*) = 8.7 × 10^14^ M^−1^ cm^−1^ nm^4^. Using the static random average *κ*^2^ of 0.69^2^, a quantum yield *Φ* = 0.6 and refractive index *n* = 1.336, we obtain a Förster radius *R*_0_ for GFP-GFP homo-FRET of 4.34 nm from the spectra, and with *κ*^2^ = 2/3, we obtain 4.59 nm, in excellent agreement with the 4.65 ± 0.09 nm quoted by (20), also using *κ*^2^ = 2/3. Homo-FRET can be investigated by steady-state anisotropy at the red edge, where the fluorophore is excited with the lowest energy. At the red excitation edge, homo-FRET is suppressed. Steady-state anisotropy measurements of eGFP and eGFP15eGFP in PBS as a function of excitation wavelength are shown in Fig. 3 *b*. The steady-state fluorescence anisotropy for monomer and dimer increased with the excitation wavelength. Both registered their lowest steady-state anisotropy values at lower excitation wavelengths in the ultraviolet region. The monomer featured higher steady-state anisotropy values compared with the dimer. This is consistent with the presence of homo-FRET for the dimer configuration, as homo-FRET provides an additional pathway for depolarization after excitation, which results in lower anisotropy values. The anisotropy response reached is maximum for the monomer from 440 to 490 nm, whereas that corresponding to the eGFP dimer remained well below this, consistent with homo-FRET. The dimer anisotropy is rising toward longer wavelengths, and at the red edge (∼510–520 nm), it is the same as that of the monomer, which is an indication of suppression of homo-FRET due to red-edge excitation.

### The inverse average fluorescence lifetime of GFP monomer and dimer scale linearly with the refractive index of their environment

**Figure 4:**
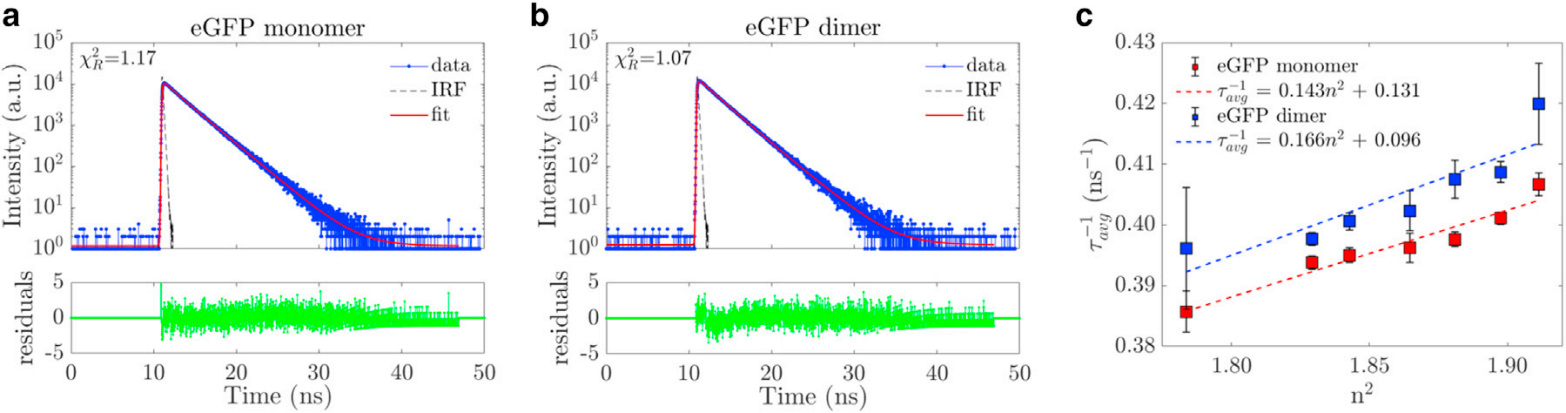
Representative fluorescence intensity decays for eGFP (*a*) monomer and (*b*) dimer in a PBS/glycerol solution with 25% glycerol. For each plot, the data are presented along with the IRF, fit, and residuals. (*c*) A plot of the inverse average fluorescence lifetime against the square of the refractive index of the solution for GFP monomer and dimer. The data for both samples are fitted showing a linear relationship as established by the Strickler-Berg formula Eq. 17. To see this figure in color, go online.

Fluorescence intensity decays were measured in a glycerol/PBS concentration range from 0 to 30% glycerol with a single detector and fitted with a double exponential decay model because a single exponential decay model did not fit well, in terms of residuals and $\chi_{R}^{2}$ ($\chi_{R}^{2}$ > 1.5). In Fig. 4, *a* and *b*, two representative intensity decays for monomer and dimer solutions in 25% glycerol/PBS are presented. Two fluorescence lifetimes were determined for each of the samples: 2.04 ns (39%) and 2.77 ns (61%) for the eGFP monomer and 2.07 ns (44%) and 2.74 ns (56%) for the eGFP dimer. The percentages in brackets denote the fluorescence intensity contributions per fluorescence lifetime component. The $\chi_{R}^{2}$ resultant from the fits were 1.17 and 1.07, respectively. Detailed data can be found in Table S1. The two fluorescence lifetimes determined in this work can be attributed to two different deprotonated excited states identified in the absorption spectrum (11,78, 79, 80).

The Strickler-Berg formula was validated by plotting the reciprocal average fluorescence lifetime of each eGFP the eGFP monomer and eGFP dimer against the square of its environmental refractive index *n* (Fig. 4 *c*). For both eGFP monomer and dimer and all the fractional solutions, the inverse average fluorescence lifetime was shown to scale linearly with the square of the refractive index (81). (This is also the case for the individual lifetimes from the double exponential fit, as shown in Fig. S2, albeit with a larger experimental uncertainty.) This phenomenon has been used as a proxy for protein concentration when observed over the cell cycle (12,13) and has shortened the GFP fluorescence decay of GFP-labeled transmembrane proteins compared with GFP-labeled proteins in the cytoplasm (82). This effect has also been used to study aerosol droplets (83).

The eGFP dimer fluorescence lifetime was slightly and consistently lower than the monomer fluorescence lifetime across the varying refractive index solutions. Whereas the monomeric eGFP only probed the solvent surrounding it, the dimeric eGFP probed its surrounding solvent and the nearby eGFP monomer along with the linker. These have a higher refractive index than the solvent, yielding a slightly shorter GFP fluorescence decay (81,84).

### Time-resolved fluorescence anisotropy measurements

#### eGFP monomer and dimer time-resolved fluorescence anisotropy decays

**Figure 5:**
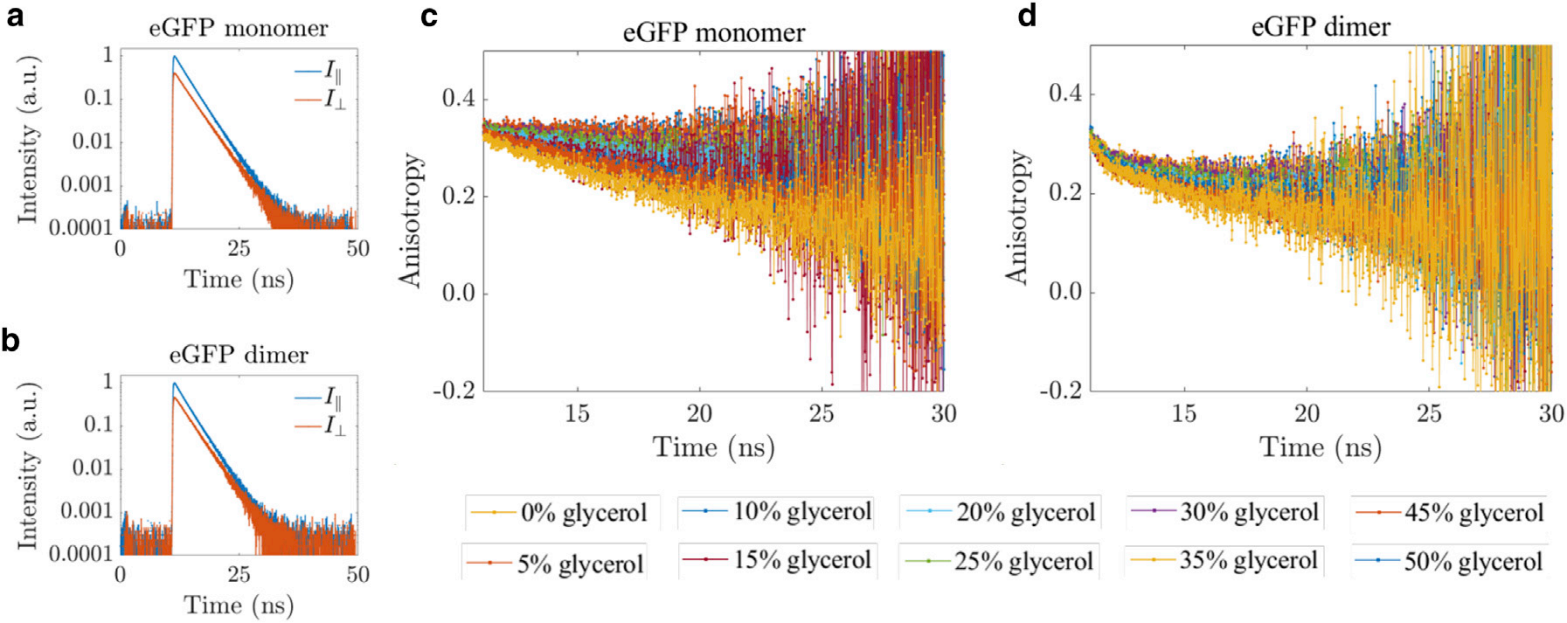
Representative parallel and perpendicular intensity decays for (*a*) eGFP monomer and (*b*) dimer. Time-resolved fluorescence anisotropy decays in different PBS/glycerol mixtures for (*c*) eGFP monomer and (*d*) eGFP dimer are given, with the glycerol content indicated. To see this figure in color, go online.

**Figure 6:**
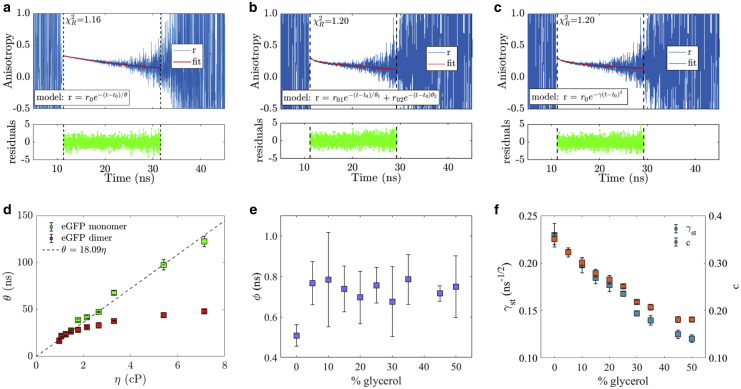
(*a*) Representative time-resolved fluorescence anisotropy decay, fit, and residuals for the eGFP monomer. The equivalent time-resolved anisotropy decay corresponding to the eGFP dimer fitted with a (*b*) double and (*c*) stretched exponential decay model. The solvent is 10% glycerol and 90% PBS. (*d*) Rotational correlation time plotted against viscosity for monomer and dimer. The monomer data are fitted with a straight line passing through the origin according to Eq. 1, with a gradient of 18.1 ns/cP. This yields a hydrodynamic radius *R*_*h*_ for the monomer of *R*_*h*_ = 2.46 ± 0.01 nm. (*e*) FRET inverse rate *ϕ* and (*f*) *γ*_*st*_ and *c* parameters are plotted against sample composition in percentage of glycerol. To see this figure in color, go online.

Time-resolved fluorescence anisotropy measurements were performed on eGFP dimers and eGFP monomers. Representative parallel and perpendicular fluorescence intensity decays for both monomeric eGFP and the dimer construct are displayed in Fig. 5, *a* and *b*. Fig. 5 *c* shows the time-resolved fluorescence anisotropy decays of the eGFP monomer in solutions of varying viscosity, and Fig. 5 *d* shows the corresponding time-resolved fluorescence anisotropy for the dimer. As expected, the eGFP monomer time-resolved fluorescence anisotropy decays follow a single exponential decay model, depicted in Fig. 6 *a*, as fluorescence depolarization only occurs through Brownian rotational motion. For eGFP in PBS, we found a rotational correlation time of 16.46 ± 0.20 ns, which agrees well with previous studies (18,37,61,80,85, 86, 87, 88, 89, 90, 91, 92). As glycerol was added, the Brownian rotational motion slowed and the rotational correlation time increased, up to 123 ± 6 ns in 50% glycerol. Because the average fluorescence lifetime of eGFP is shorter than the rotational correlation time (and even decreases slightly as glycerol is added; see Fig. 4 *c* and Table S1), it became increasingly difficult to measure it, and the experimental uncertainly of eGFP’s rotational correlation time increased.

The eGFP dimer time-resolved fluorescence anisotropy decays were not appropriately fitted with a single exponential decay model because a very short component was identified at the beginning of the decay, a typical signature of homo-FRET between fluorescent proteins (27). To fit the eGFP dimer anisotropy data, two decay models were used: a double exponential (Eq. 13) and a stretched exponential (Eq. 10; Fig. 6, *b* and *c*). Both models assume that the protein configuration is isotropic and randomized, without any preferential orientation within the solution (29,30,32,63) and *r*_∞_ = 0. The double exponential decay model accounts for a slow Brownian rotational motion (with the rotational rate of the protein in the excited state much lower than the radiative and nonradiative de-excitation rates) and fast homo-FRET (29,30). The stretched exponential decay model accounts for a distribution of rate constants for depolarization (32,63). The double exponential decay model has six free fitting parameters (background, shift, two pre-exponential factors, and two decay times), whereas the stretched exponential only has five (background, shift, pre-exponential factor, dimensionality, and γ).

#### The double exponential decay model fails to explain the eGFP dimer dynamics

The rotational correlation times calculated from the fit of the eGFP dimer anisotropy data with the double exponential decay model (Eq. 13) and in solutions of varying viscosities were compared with those from the eGFP monomer (Fig. 6 *d*; Table S2). The rotational correlation times of the monomer (*green squares*) were plotted versus the solution viscosity and fitted with a straight line. The gradient is 18.1 ns/cP and goes through zero as demanded by Eq. 1. From the fit, the hydrodynamic radius of the eGFP was calculated: *R*_*h*_ = 2.46 ± 0.01 nm. This is in good agreement with values previously reported (93, 94, 95, 96).

The rotational correlation times of the eGFP dimer were shown to follow a similar trend for low viscosity values, up to ∼2 cP (Fig. 6 *d*; Table S3) but then leveled off for viscosity values larger than 2 cP and showed an apparent lower rotational correlation time in comparison to the monomer (*red data points*). The shorter FRET inverse rate *ϕ* presented no correlation with viscosity (Fig. 6 *e*). A shorter rotational correlation time of the dimer compared to the monomer would imply that the dimer rotates faster than the monomer in a solvent of the same viscosity. This is impossible because its radius of gyration is larger.

This means that the fit parameter associated with the long rotational correlation time appears to include a combination of Brownian rotational motion and FRET. The rotational correlation time of eGFP15eGFP should be larger than the corresponding one for the monomer, as shown by the MD simulations discussed below.

#### FRET efficiency heterogeneities between eGFPs can produce multiexponential anisotropy decays

The logic used behind the application of the stretched exponential decay model lies in considering a distribution of distances and orientations between the FRET pairs. In the dynamic averaging regime in which the rotational rate of the fluorophore in the excited state is much higher than its fluorescence decay rate, the average orientation per FRET pair is given by $\langle \kappa^{2}\rangle$. However, here, for a fluorophore as large as GFP, 〈|κ|〉 determines the average orientation between the transition dipole moments of the donor and acceptor. When the donor's fluorescence decay is much shorter than its rotational rate, the system behaves in the static averaging regime. Thus, each eGFP separation and orientation per FRET pair would contribute as a single component within the time-resolved fluorescence anisotropy decay, yielding a multiexponential decay.

The eGFPs are considered to be motionless during the timescale of the fluorescence decay because the fluorescence lifetime of eGFP is much shorter (∼2.5 ns (11,78, 79, 80); Table S1) than its rotational correlation time (∼15 ns in water (18,37,61,80,85, 86, 87, 88, 89, 90, 91, 92); Table S2). Therefore, the fluorescence anisotropy is assumed to be exclusively determined by the process of energy transfer. Because the sample consists of a diluted solution of eGFPs, we can model it as a spatially isotropic and disordered system. Moreover, the concentration of proteins is sufficiently low that the probability of multimigration processes is negligible and all the pairwise interactions between the excited donor and acceptor are independent. In addition, the initially excited eGFP (donor) is considered to be the major contributor to the anisotropic response, and a three-dimensional solution was initially considered by setting *δ* = 1/2 in Eq. 10 (31,32,63,64). This reduced the number of free fitting parameters to four, the same as for a single exponential anisotropy decay (Eq. 9). The fit of the eGFP dimer anisotropy data with the stretched exponential decay model yielded two fit parameters: *r*_0_ and *γ*_*st*_. The fit parameter *γ*_*st*_, given by Eq. 11, decreased with the solution viscosity (Fig. 6 *f*), whereas *r*_0_ presented no correlation (see Table S3).

From Eq. 11, *γ* is expected to vary slightly with the refractive index of the solution because of its dependence on the lifetime, which is a function of the refractive index, but not quite as much as we observe. According to theory, when the system is isotropic and three-dimensional and behaves within a static regime, the orientation factor |*κ*| can be averaged to 0.69 (64,97,98) and is thus constant. The dimensionless parameter *c* should also be constant but varies too, as shown in Fig. 6 *f*, with the difference between *γ* and *c* due to the refractive index effect on the average GFP lifetime. One possible explanation for the larger decrease of *γ*_*st*_ than expected and the variation of *c* could be related to a small contribution from Brownian rotational motion, which depolarizes the emission and is not fully accounted for in the stretched exponential model. In the low viscosity region, depolarization due to rotational motion will be added to depolarization due to homo-FRET and thus inflate these values. This is less of an effect in higher-viscosity regimes.

**Figure 7:**
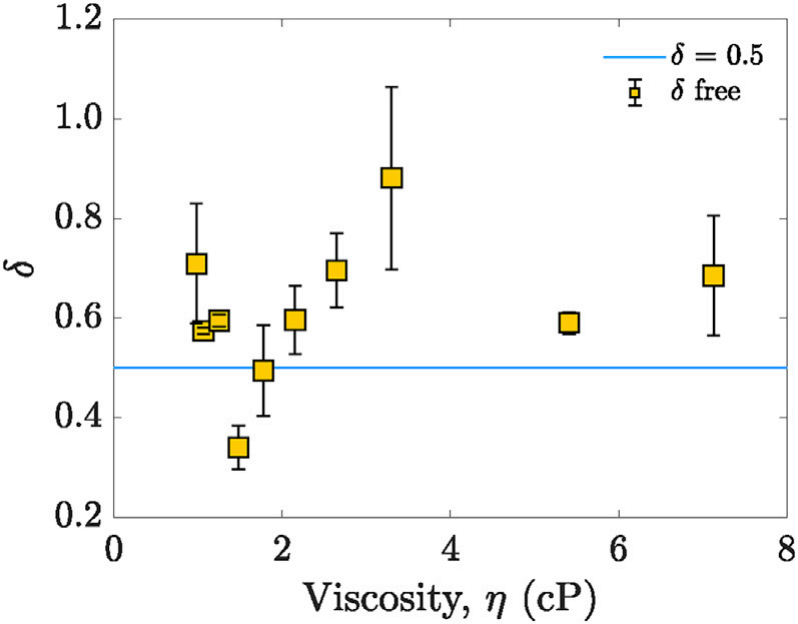
The dimensionality parameter *δ* from the stretched exponential decay model (Eq. 10), allowed to float freely in the fit, plotted against the solution viscosity. *δ* = 0.5, which indicates a three-dimensional system, is given by the cyan continuous line. To see this figure in color, go online.

To validate *δ* = 0.5, the anisotropy data were also fitted with the stretched exponential decay model, letting *δ* be a floating fit parameter. Values of *δ* very close to 0.5 were found across all the varying viscosity solutions (see Fig. 7 and Table S4), which confirms that considering the system as three-dimensional is a sensible approach.

On an additional note, it is worth mentioning that the goodness of the stretched exponential decay fit given by $\chi_{R}^{2}$ was the same for the double exponential decay model, which has more fitting parameters, even when *δ* is not fixed at 0.5 and is a floating fit parameter. Thus, the double exponential decay model is not a statistically justified model.

#### Experimental E_FRET_ per eGFP dimer anisotropy model

FRET theory was applied to calculate the FRET efficiencies (Eqs. 12 and 14) when the anisotropy data were interpreted via stretched exponential (31,32) and double exponential (30) decay models. For this purpose, the experimental average fluorescence lifetime *τ* of the single eGFP monomer was employed. Detailed data are given in Table S5.

**Figure 8:**
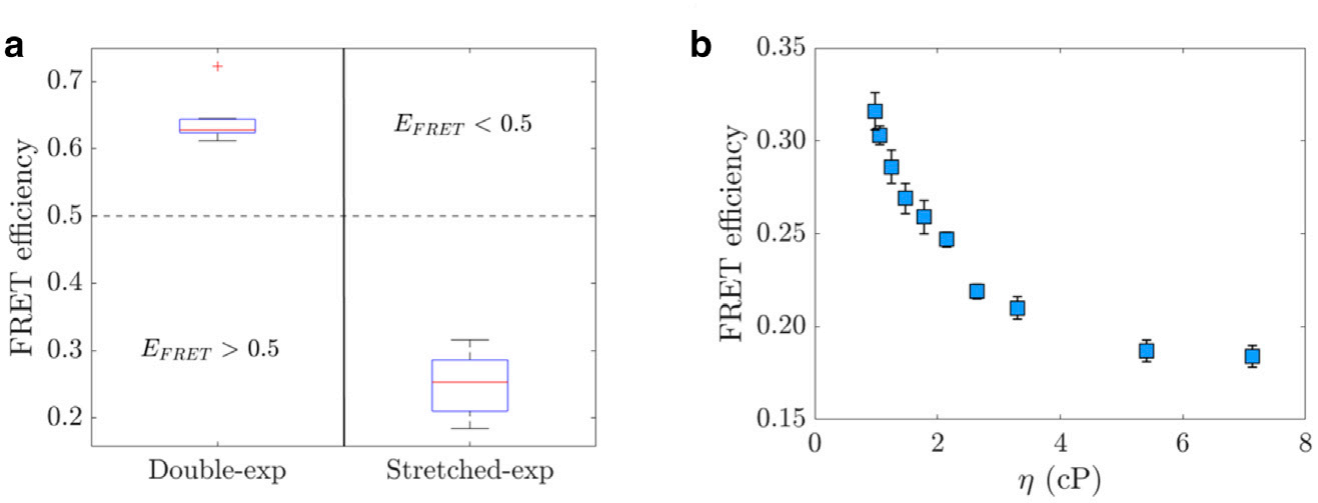
(*a*) Boxplots of the calculated FRET efficiencies between the two eGFPs of the dimer for each anisotropy decay model: double and stretched exponential. The horizontal red line in the boxplot corresponds to the median, and the bottom and top blue edges of the box correspond to the 25th and 75th percentiles. The whiskers extend to the most extreme data points not considered outliers, and the outliers are plotted individually using the “+” symbol. (*b*) Relationship between *E*_*FRET*_ and the solution viscosity. To see this figure in color, go online.

Fig. 8 *a* shows that the FRET efficiencies calculated from the double exponential decay model (Eqs. 13 and 14) were located within a narrow range above 0.5 (between 0.617 ± 0.002 and 0.723 ± 0.001, in 50% glycerol solution and PBS, respectively). No correlation between FRET efficiencies and solution viscosities were encountered. This was an expected result because the fluorescence lifetime only varied by ∼9% (between 2.65 ± 0.01 ns in PBS, and 2.41 ± 0.02 ns in 50% glycerol solution) across solutions (Table S5), and the FRET inverse rate *ϕ* showed no correlation with the solution viscosity (Fig. 6).

The FRET efficiencies calculated from the stretched exponential decay model (Eq. 12) were below 0.5 (between 0.18 ± 0.01 and 0.32 ± 0.01 in 50% glycerol solution and PBS, respectively) and within a much wider range of values, presenting a correlation with the solution viscosity in the same way as the *γ*_*st*_ parameter (higher values were associated with low viscous solutions and lower values with high viscous solutions) (Fig. 8 *b*). As pointed out in the previous section, the *γ*_*st*_ parameter should remain largely unchanged, which would result in a narrower range of FRET efficiencies. However, residual Brownian motion may contribute to depolarization, which may inflate FRET efficiencies, particularly in the lower viscosity regime, and spread out the FRET efficiency range.

Because the distance between proteins and their relative orientations cannot be separated experimentally, MD simulations were performed to investigate these parameters independently.

### Molecular dynamics simulations

#### Molecular dynamics simulations: orientation factors

FRET efficiencies were calculated from the MD simulation trajectories (*κ*^2^ and *R*) by combining Eqs. 6 and 7, with *J*(*λ*) = 8.7 × 10^14^ M^−1^ cm^−1^ nm^4^, *n* = 1.336, and *Φ* = 0.6.

**Figure 9:**
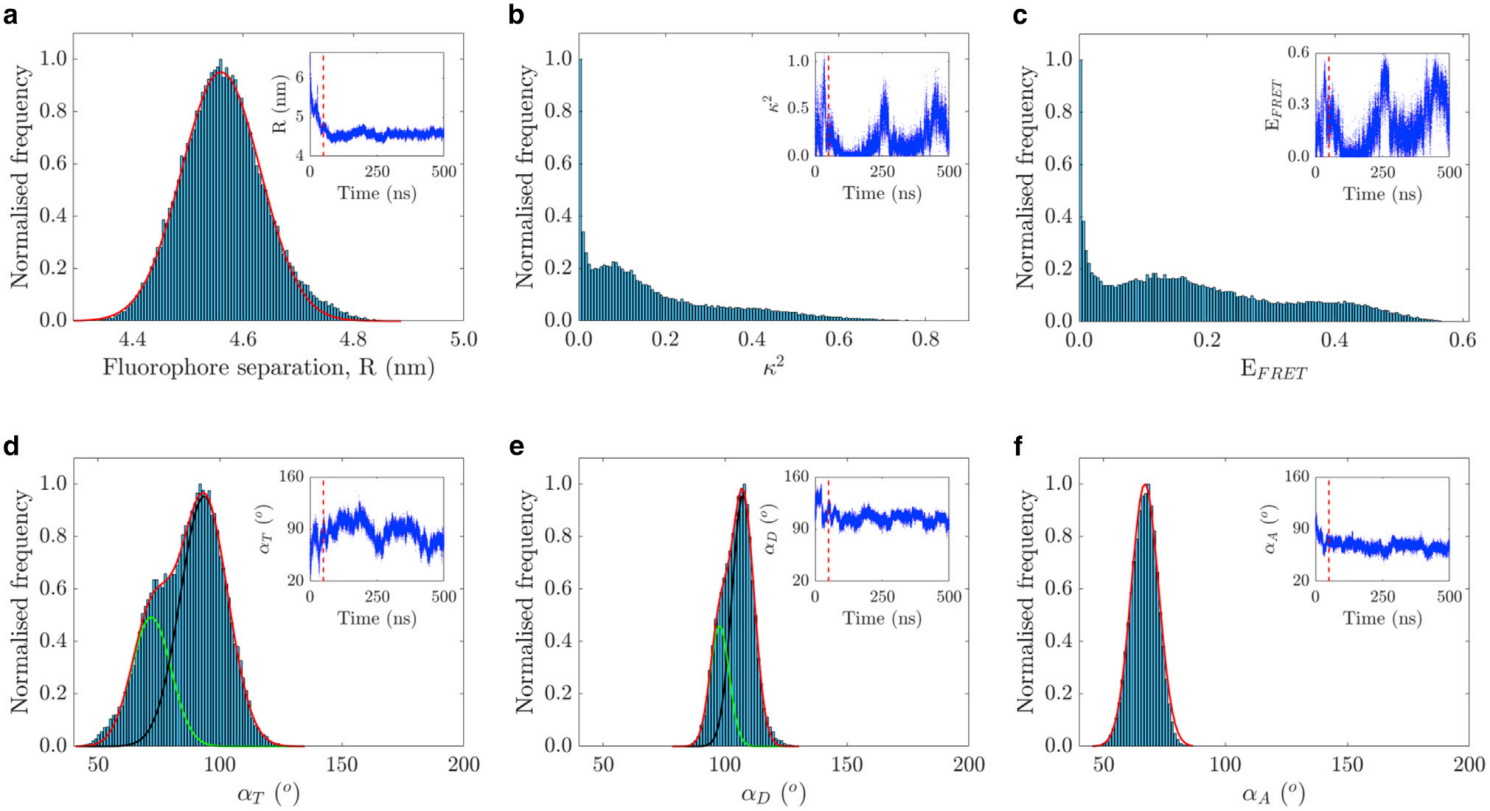
Distributions and temporal evolution (*inset*) from MD simulations with sampling every 10 ps of (*a*) fluorophore separation *R*, (*b*) relative dipole orientation *κ*^2^ between GFP monomers, (*c*) FRET energy efficiency *E*_*FRET*_ calculated from Eq. 6 with *R*_0_ = 4.65 nm, (*d*) angle between GFP monomers *α*_*T*_, (*e*) angle between one GFP monomer and the vector $\vec{R}$, and (*f*) angle between the other GFP monomer and $\vec{R}$. Single and double Gaussian functions were used to fit the separation and angular distributions, with further details given in the main text. Dashed vertical red lines shown in the inset plots define the starting point to generate the distributions. To see this figure in color, go online.

The distributions of *R* and *κ*^2^ obtained from the last 450 ns of the MD simulations are shown in Fig. 9, *a* and *b*, respectively. In the inset plots, their temporal evolutions are shown, with the red dashed line indicating the starting point for collecting statistics for the corresponding distribution. *R* was approximately constant over time, and a Gaussian fit of its distribution (*red line*) yielded 〈R〉 = 4.56 ± 0.07 nm (Fig. 9 *a*). However, *κ*^2^ presented an asymmetric and broad distribution, whereas its temporal evolution showed a well-defined and repetitive pattern (Fig. 9 *b*). Because *R* is approximately constant, the variation in *κ*^2^ is mirrored by the variation in *E*_*FRET*_ (Fig. 9 *c*).

The *κ*^2^ histogram shows a range of values between 0 and 0.918, with a peak at *κ*^2^ = 0, gradually dropping to zero for *κ*^2^ = 1. *κ*^2^ = 1 implies that the transition dipole moments of both monomers belong to parallel planes and are perpendicular to the vector that defines their separation, $\vec{R}$. Because the transition dipole moment is defined within a plane perpendicular to the main axis of the GFP *β*-barrel (Fig. 2), the two monomers would be perfectly stacked on top of each other. The rest of the *κ*^2^ distribution (0 ≤ *κ*^2^ < 1) was due to tumbling and rotation around the previously described aligned orientation. Because most *κ*^2^-values within this range were closer to 0 than to 1, the preferred configuration of the two GFPs was with the two transition dipole moments almost perpendicular to each other. For *κ*^2^ between 0 and 1, two broad peaks can be observed around ∼0.1 and 0.45. These populations are easier to discern in the FRET efficiency probability distribution, which extends from 0 to almost 0.6 (Fig. 9 *c*).

A rapid rotation took place in the first 50 ns, supported by the decrease on the fluorophore separation *R*, with a concomitant twist of the linker (Fig. 9 *a*). From the first 50 ns onward, the fluorophore separation remained approximately constant, and *α*_*T*_ oscillated around the perpendicular configuration, *α*_*T*_ = 90° (Fig. 9 *d*). Specifically, the probability distribution of the angle *α*_*T*_ was fitted with a double Gaussian function, centered at 72 ± 8° (*green line*) and 94 ± 10° (*black line*), where a greater amount of events was found in the second peak. Additionally, smooth tumbling and rotation were supported by the calculation of the angles *α*_*D*_ and *α*_*A*_, whose values were in close proximity to 90°, either above (*α*_*D*_) or below (*α*_*A*_) this value (Fig. 9, *e* and *f*). Like the *α*_*T*_ probability distribution, the one corresponding to the angle *α*_*D*_ was fitted with a double Gaussian function, with a second predominant peak of events. The two Gaussian functions were centered at 98 ± 4° (*green line*) and 108 ± 4° (*black line*). The angle *α*_*A*_ distribution was fitted with a single Gaussian-function, with $\alpha_{A}=67\pm 5{}^{\circ}$ (*red line*). For the first two angular distributions (*α*_*T*_ and *α*_*D*_), the convolution of the two Gaussian fit functions is shown as a red envelope. To visualize the *κ*^2^ and angular distributions analysis, a video of the temporal evolution of the two GFPs tethered by the 15-amino-acid linker is available as Video S1, together with scatter plots showing the relative orientation of the two GFPs transition dipole moments (Fig. S3).

Video S1. Simulated Video of the GFP Dimer

The low magnitude of *κ*^2^ (<0.8, Fig. 9 *b*) indicates that the two proteins arrange themselves such that they do not fully and homogeneously explore all possible orientations. This is not unexpected because the linker restricts the full range of all possible orientations available to the two GFPs. The broad and asymmetric distributions of *κ*^2^ and *E*_*FRET*_ have a large standard deviation, in agreement with other work (99), which, along with their mean values, was given by $\langle \kappa^{2}\rangle$ = 0.17 ± 0.16 and $\langle E_{FRET}\rangle$ = 0.18 ± 0.14, respectively. Because the vast majority of the FRET efficiencies calculated from the MD simulations lie below 0.5, the results obtained from the stretched exponential decay model (Eq. 12) are closer to the MD simulations than the calculated ones via the double exponential decay model (Eq. 14; Fig. 8 *a*).

**Figure 10:**
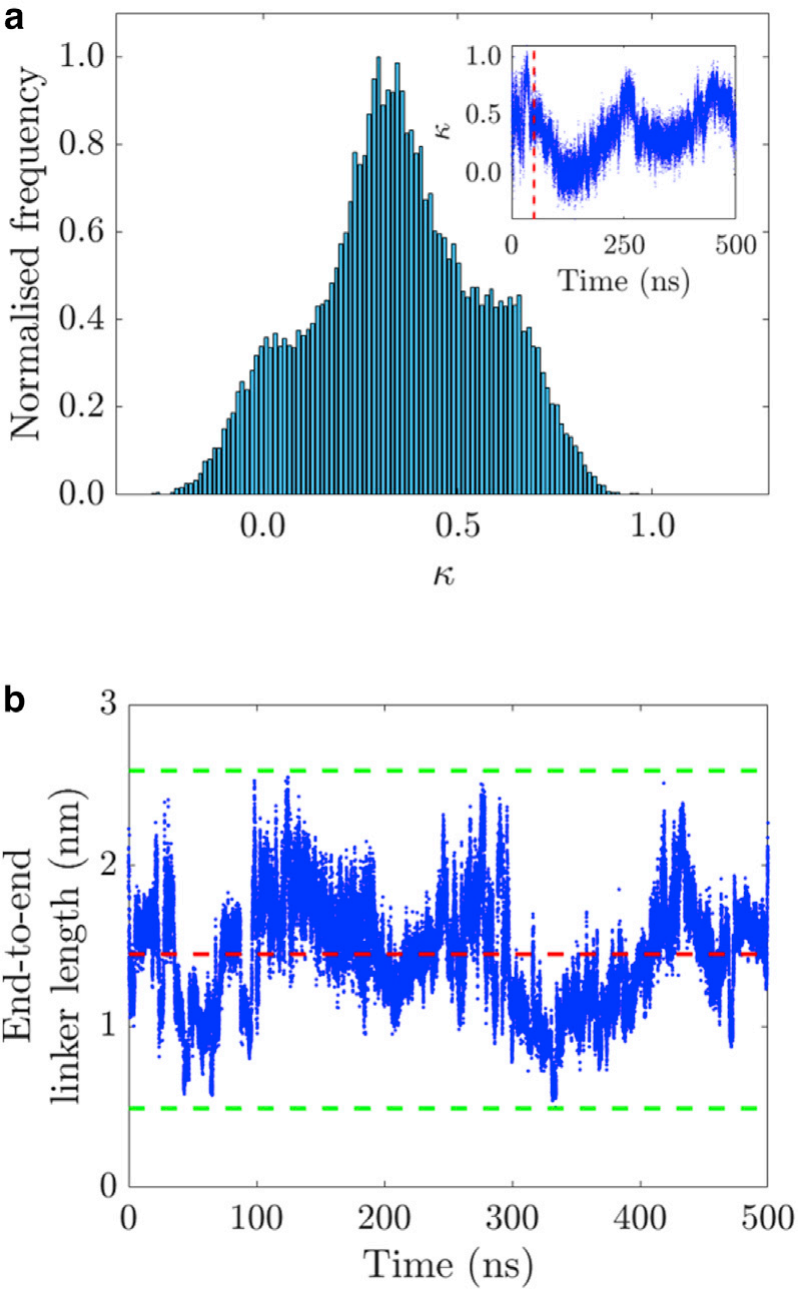
(*a*) Distribution and temporal evolution (*inset*) from MD simulations with 10 ps sampling of the orientation factor *κ*. The dashed vertical red line in the inset indicates the start of the distribution. (*b*) Temporal evolution of the end-to-end linker length, in which the horizontal dashed red line corresponds to the mean value (1.45 nm) and the bottom and top dashed green lines refer to the minimal and maximal values (0.49 and 2.59 nm, respectively). To see this figure in color, go online.

The *κ* distribution obtained from the simulations was calculated and is shown in Fig. 10 *a*, in which the inset plot corresponds to its temporal evolution over the duration of the simulation. The distribution was calculated from the data points after the dashed vertical red line at t = 50 ns. Equation 4 was employed to calculate *κ*, except for the square exponent. Three predominant populations of *κ* were discernible from its distribution from around −0.4 to 1.1. The most frequent values were at around 0.4 and formed the central peak of the distribution, with a smaller peak at 0.05, and a shoulder at 0.6. The majority of *κ*-values were located above 0, with very few in the negative range of the distribution. Therefore, *κ* and |*κ*| mean values are expected to be almost identical. The mean of the absolute value of *κ* was calculated, yielding 〈|κ|〉 = 0.35 ± 0.20. Because this result is well below the theoretical 0.69 for the static random averaging regime (64,97), this further shows that the two proteins are not exploring all the available three-dimensional space, with the 15-amino-acid linker restricting their mobility.

Moreover, the distance from end to end of the linker was investigated over time. This is presented in Fig. 10 *b*, in which the data oscillate around a mean value of 1.45 ± 0.34 nm (*red dashed line*), from 0.49 to 2.59 nm (*bottom* and *top green dashed lines*). Because the linker is ∼6 nm long when fully stretched and the graph places its end-to-end mean value around 1.45 nm, the linker is significantly coiled. It seems reasonable to think that the flexibility of the linker is an intrinsic property of itself instead of considering any dependence with the viscosity of the medium. Thus, we assumed that the different orientations established by the two GFPs and given by the orientation factor *κ*^2^ would be invariant in outcome but enriched from a statistical point of view with a decrease in the environmental viscosity, collecting the same *κ*^2^ data in a shorter amount of time. Even if this were not the case, we would not be able to prove it experimentally because of the short fluorescence lifetime associated with these slow-motion fluorophores. Nonetheless, we believe that *κ*^2^ should depend on the linker length rather than on the solution viscosity. A longer linker should confer a wider distance range on this FRET pair and most likely also a wider *κ*^2^ range. Conversely, some restriction of the linker may lead to a stronger restriction of the *κ*^2^ orientations and thus to a more pronounced skewness of its distribution in comparison to randomly orientated GFPs (99).

It is worth mentioning, however, that a longer simulation time may in principle reveal a more complex behavior of the linker. By construction, the configurations characterized by a fully stretched linker would not be accessible because of its initial folding and the subsequent system solvation and unit cell definition, although they would be expected to be rare events. Although we are not able to assess with certainty that no other metastable energetic minima could be observed at unexplored points in the space defined by *κ*^2^ and *R*, the tendency of the linker to fold, which dragged the two monomers close to one another within the first 50 ns, allows us to reasonably assume that the configuration observed beyond 50 ns is the absolute energetic value.

Overall, under the assumption that the MD simulations represent the absolute energetic minimum in the space defined by the orientational angles *α*, orientation factor *κ*, and the separation between the fluorophores $\vec{R}$, the system would be in a highly restricted static averaging regime. The agreement between anisotropy decay analysis based on the stretched exponential function and simulation may constitute an a posteriori validation of the initial assumption.

#### Time-resolved anisotropy decays of GFP and GFP15GFP in water from molecular dynamics simulations

**Figure 11:**
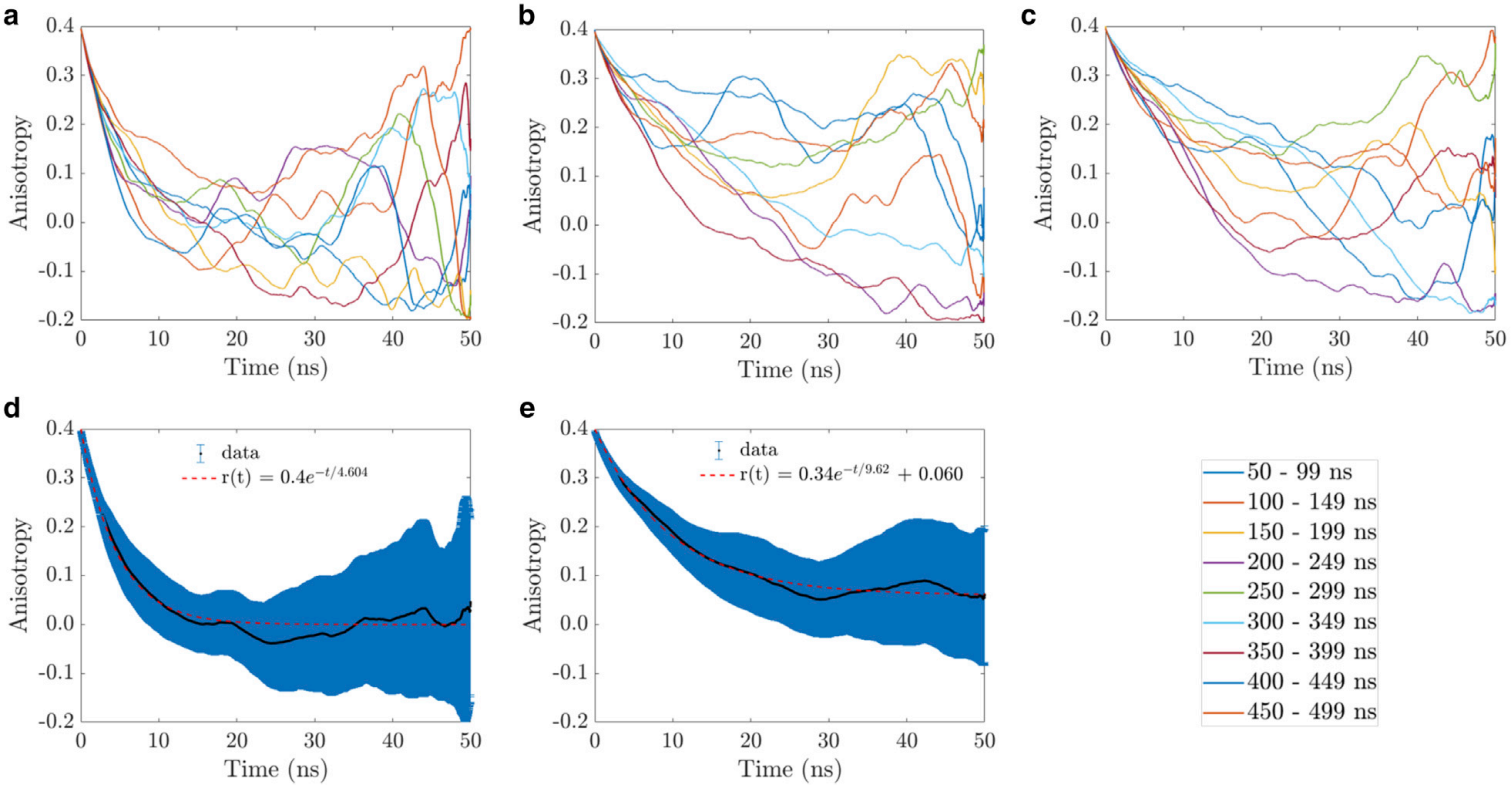
(*a*) Individual simulated anisotropy decays of the GFP monomer and (*b* and *c*) each GFP monomer of the GFP15GFP dimer, in water. (*d*) Average simulated anisotropy decay (*solid black line*) of the monomeric GFP, with fit (*dashed red line*). (*e*) Average simulated anisotropy decay of the GFP15GFP dimer, with fit. The blue areas of (*d*) and (*e*) are error bars that account for the calculated standard deviation per data point. The legend of the nine individual anisotropy decays from the 500 ns simulation of (*a*)–(*c*) is located in the lower right corner. To see this figure in color, go online.

To assess the rotational mobility of the GFP monomer and the GFP15GFP dimer, individual autocorrelation curves—which represent fluorescence anisotropy decays—were generated every 50 ns with a time resolution of 10 ps according to Eq. 3, averaged, and fitted. Fig. 11 *a* shows nine individual anisotropy decays for the GFP monomer from nine 50 ns time windows of the 500 ns simulation, and Fig. 11, *b* and *c* correspond to nine anisotropy decays for each GFP in the GFP15GFP dimer. An average anisotropy decay for the GFP monomer is shown in Fig. 11 *d*, and the equivalent for the dimer in Fig. 11 *e*. Both average anisotropy decays were fitted under the assumption that the anisotropy depolarization was strictly governed by the Brownian rotational motion, in which no FRET was present.

For the GFP monomer, the average time-resolved anisotropy decay was very well fitted with a single exponential decay model (Fig. 11 *d*), which assumes that the protein is freely rotating and its shape can be modeled as a sphere. To assess how good this spherical approximation was, the anisotropy decay was also fitted with a double exponential decay model, giving rise to two identical rotational correlation times (data not shown). This means that reducing the structure of GFP to a sphere is a valid approach. The rotational correlation time extracted from the single exponential decay fit was 4.60 ± 0.04 ns, with a goodness of fit of *R*^2^ = 0.98. This low value for the rotational correlation time in comparison with the experimental rotational correlation time in Fig. 6 *d* (16.5 ± 0.2 ns) is due to the TIP3P water model used in the MD simulations. In fact, the viscosity of TIP3P water is 0.321 cP at room temperature (52, 53, 54), considerably lower than the experimental water viscosity (1 cP). Although the experiments were measured in PBS buffer, its viscosity was considered to be identical to water (100,101). With this information and applying the Stokes-Einstein-Debye equation (Eq. 1), the actual rotational correlation time of the GFP monomer in water can be recalculated by multiplying the result of the simulation by a scaling factor 1/0.321, which yields 14.3 ± 0.1 ns. This scaled value is in reasonable agreement with the experimentally measured rotational correlation time, *θ* = 16.5 ± 0.2 ns (18,85, 86, 87).

For the GFP15GFP dimer, the data were initially fitted with a double exponential decay model, in which one component presented a much longer rotational correlation time compared to the other. Then the contribution of this component was reduced to *r*_∞_, yielding a hindered rotation model or the so-called wobble-in-a-cone model. The fit parameters and goodness of fit were found to be *r*_0_ = 0.398 ± 0.001, *r*_∞_ = 0.0604 ± 0.0003, *θ* = 9.62 ± 0.05 ns, and *R*^2^ = 0.98. *r*_∞_/*r*_0_ = 0.152, and the interpretation of this model, according to Eq. 19, is that each GFP wobbles around a common axis with a cone semiangle of 59.03° (72,73) while the entire entity (GFP15GFP) rotates slowly. The GFP dimer tumbling in such a restricted way is consistent with the narrow distribution of the orientation factor *κ*^2^ in the FRET simulations and is analogous to a fluorophore with restricted rotation in an ordered lipid bilayer (72). The scaled rotational correlation times of the two GFP constructs in PBS are 30.0 and 14.3 ns for dimer and monomer, respectively. These results confirm the GFP dimer rotates slower than the monomer and thus rules out the validity of the double exponential decay model to interpret the experimental time-resolved fluorescence anisotropy data.

In experiments, when the fluorescence lifetime of a large and thus slowly rotating protein is very short, the calculation of its rotational correlation time can be very challenging and inaccurate. This is why for large proteins, a probe with a longer fluorescence lifetime should be employed (102). We note that because MD simulations only account for dynamical properties via the autocorrelation function in Eq. 3 and do not involve any fluorescence emission, there is no restriction coming from the fluorescence lifetime. In general, this allows the rotational correlation to be calculated independent of the fluorescence properties of the probe.

## Conclusions

In this work, we studied homo-FRET using a new, to our knowledge, homo-FRET standard, eGFP15eGFP, formed by two eGFPs tethered by a linker of 15 amino acids (Fig. 2). Steady-state anisotropy with red-edge excitation shows that homo-FRET occurs, and time-resolved fluorescence anisotropy experiments, analyzed with a stretched exponential decay model, allow us to calculate a FRET efficiency of around 25% for the eGFP dimer construct. The fluorescence decays of both the GFP monomer and dimer are a function of the refractive index of their environment, as observed previously (11).

Although in experimental FRET work, the distance and orientation of the fluorophores cannot be separated, MD simulations allow the distinction between distance and orientational contributions in the FRET efficiency. MD simulations revealed the distribution of the orientation factor *κ* via the angular distributions of the vectors separating the fluorophores (*α*_*T*_, *α*_*D*_, and *α*_*A*_, as defined in Fig. 2), as shown in Fig. 9, *b* and *d*–*f*. The two GFPs orientate themselves within a constrained frame, in which tumbling and in-axis rotation of each individual GFP *β*-barrel equally contribute to the restricted range of orientational angles and thus of *κ* and *κ*^2^. The fluorophore distance does not show significant variations (see Fig. 9 *a*), and together with *κ*^2^, this allows us to calculate an FRET efficiency of $\langle E_{FRET}\rangle$ = 0.18 ± 0.14, from Eq. 6, which agrees well with the experimental data. Moreover, the autocorrelation of the tumbling motion enables us to calculate the equivalent of a fluorescence anisotropy decay, which also agrees well with the experimental data.

Thus, the combination of time-resolved fluorescence anisotropy and MD simulations allows us to gain detailed insight into the behavior of the GFP dimer. We propose this construct as a homo-FRET standard to be used for comparison with homo-FRET-based biosensors (103) and potentially as a reference when studying dimerization processes in cells via FRET between GFP.

The simulation data supporting this research are openly available from the King’s College London research data archive at http://doi.org/10.18742/RDM01-638.

## Author Contributions

Y.T.-G. and K.S. designed the research. Y.T.-G. acquired the experimental data, and A.C. and C.M. designed and performed the MD simulations. A.J.B. and R.L.B. created the eGFP standards and helped with the design of the MD simulations. The experimental and simulated data were plotted and analyzed by Y.T.-G., who also wrote this article. A.L.M. helped with the experimental setup and data analysis and J.N. critically reviewed and edited the published work. All authors reviewed the manuscript.

## Supporting Material

Document S1. Supporting Materials and Methods, Figs. S1–S3, and Tables S1–S5

Document S2. Article plus Supporting Material
