# Supplementary material for: Time-Resolved Fluorescence Anisotropy and Molecular Dynamics Analysis of a Novel GFP Homo-FRET Dimer
Source: Biophys J. 2020 Dec 18;120(2):254–69. doi: 10.1016/j.bpj.2020.11.2275 (PMC7840444; doi:10.1016/j.bpj.2020.11.2275)
Supplement: Document S1. Supporting Materials and Methods, Figs. S1–S3, and Tables S1–S5 [file mmc1.pdf]

Biophysical Journal, Volume *120*

Supplemental Information

Time-Resolved Fluorescence Anisotropy and Molecular Dynamics Analysis of a Novel GFP Homo-FRET Dimer

Yurema Teijeiro-Gonzalez, Alessandro Crnjar, Andrew J. Beavil, Rebecca L. Beavil, Jakub Nedbal, Alix Le Marois, Carla Molteni, and Klaus Suhling

SUPPLEMENTARY MATERIAL

1. Theoretical background of the stretched exponential decay model

Although the derivation of the stretched exponential decay model has been covered in the literature, we briefly outline it since the procedure gives insight into the modelling of the system.

In solutions with motionless fluorophores during their excited state ($\tau \ll \theta$), the fluorescence anisotropy depolarisation can be thought to be exclusively dependent on FRET, whose rate decreases when increasing the separation between fluorophores (1).

When the fluorophores are identical, in addition to direct excitation of the donor (initially excited fluorophore), there is also a probability of re-excitation of the donor by the acceptor. The initially excited fluorophore is known to be the major contributor to the fluorescence anisotropy, since the fluorescence emission of excited fluorophores due to FRET depolarises nearly completely. The probabilities of these events to happen and contribute to the fluorescence anisotropy depolarisation are determined by the probability $H(t)$(2). Thus, the fluorescence anisotropy takes the form:

$$r(t) = r_0 H(t) \tag{S1}$$

where r_0 is the initial anisotropy at time $t = 0$. The probability $H(t)$ was calculated applying the Huber-Hamilton-Barnett method(3). In order to determine it, one must start from the excitation probability of the initially excited fluorophore (donor) as a combination of rate equations:

$$\frac{dP_k(t)}{dt} = -\sum_l \omega(R)\big(P_k(t) - P_{k'}(t)\big) \tag{S2}$$

where the radiative decay term given by the fluorescence lifetime τ is omitted from the right-hand side as the overall fluorescence lifetime remains invariant due to homo-FRET. $P_k(t)$ is the probability of the donor to be excited at time t and $P_{k'}(t)$ is the probability of an acceptor to be excited by the donor at time t. The term $\omega(R)$ refers to the transfer of energy among fluorophores (FRET rate constant), identical in both directions ($\omega(R)_{kk'} = \omega(R)_{k'k}$) and is given by the following expression:

$$\omega(R) = \frac{1}{\tau}\frac{3}{2}\kappa^2(\Omega)\left(\frac{R_0}{R}\right)^6 \tag{S3}$$

where τ is the isolated donor fluorescence lifetime, R_0 corresponds to the Förster distance, distance at which the energy efficiency due to FRET is half, and $\kappa^2(\Omega)$ describes the dipole interaction between donor and acceptor as a function of their relative orientation.

The first term on the right-hand side of equation S2 describes the loss of nonradiative energy of the initially excited fluorophore (donor) via FRET. The second term describes the re-excitation of the initially excited fluorophore (donor) due to the back transfer from the acceptor(3, 4).

Let's now consider the case of a single donor and a single acceptor. The donor will be designated with the subscript 1 ($k \to 1$, from equation S2) and the acceptor with the subscript 2 ($k' \to 2$). Setting the initial conditions $P_1(0) = 1$ and $P_2(0) = 0$, equation S2 can be solved for both fluorophores (donor and acceptor). The solution for the donor, which is the one we are interested in, is as follows:

$$P_1(t) = \frac{1}{2}\big(1 - e^{-2\omega(R)t}\big) \tag{S4}$$

When the system consists of an array of fluorophores (donors and acceptors), the total fluorescence anisotropy will be given by all the donors' contributions. For this reason, the donor's excitation probability given by equation S4 must be expanded over the entire system, which gives rise to the configurational average $H(t)$.

If the system is spatially conformed by an ordered array of fluorophores, such as a lattice, the transfer of energy will be identical everywhere. However, if this array is not ordered, the transfer of energy via FRET will depend on the local fluorophore concentrations and thus on the fluorophore separations. A spatial disorder, such as a diluted system, is modelled in such way that the donor occupies a central position at the origin and only independent pairwise interactions (donor and acceptor) are assumed to take place. If the fraction of sites occupied at random by fluorophores is given by p and the fraction of unoccupied sites as $1 - p$, then the configurational average $H(t)$ takes the following form(3, 5):

$$H(t) = \prod_{i=1}^{N}[pP_i(t) + (1-p)] = exp\left(\sum_{i=1}^{N} ln[1 + p(P_i(t) - 1)]\right) \tag{S5}$$

where $P_i(t)$ corresponds to the i_{th} donor's excitation probability over the N lattice sites. This expression immediately implies that two fluorophores cannot occupy the same lattice site.

To indicate that the number of fluorophores within the lattice is low, the approximation $p \ll 1$ is introduced in equation S5. This approximation neglects the possibility of encountering two donors in the same neighbourhood. Thus, equation S5 can be rewritten as follows(3, 5):

$$H(t) \approx exp\Big(-p\sum_{i=1}^{N}(1-P_i(t))\Big) \tag{S6}$$

If we introduce the excitation probability of each individual donor $P_i(t)$ (equation S4) in equation S6, and the summation over sites is replaced by an integration over space, in a logarithmic scale, $H(t)$ takes the form(2, 5):

$$lnH(t) \approx -\frac{\rho}{2}\int_0^{\infty}\Big(1-e^{-2\omega(R)t}\Big)u(R)dR \tag{S7}$$

where the probability p becomes a number density ρ and $u(R)$ represents a continuous/uniform spatial distribution. In a 3-dimensional scenario $u(R) = 4\pi R^2$.

When the rotational rate of the fluorophore is much higher than the FRET rate between fluorophores, the fluorescence anisotropy of the overall system can still be assumed to decay only due to FRET. The fluorophores are still in the same position (no translational diffusion) and due to rapid rotation, the rotational correlation time θ is too small to be taken into account in the fluorescence anisotropy decay. In this case the system is said to be in the dynamic averaging regime and $\kappa^2(\Omega)$ can be averaged as follows(5, 6):

$$\langle\kappa^2\rangle = \int_{\Omega}\kappa^2(\Omega)\nu(\Omega)d\Omega \tag{S8}$$

where $\nu(\Omega)$ is the angular distribution for an isotropic system(5).

Thus, $\kappa^2(\Omega)$ is replaced by $\langle\kappa^2\rangle$ in the transfer rate expression (equation S3). If we solve equation S7 for a 3-dimensional scenario and introduce in equation S1, the fluorescence anisotropy takes the following form:

$$r(t) = r_0 e^{-\frac{\rho}{2}\frac{4\pi\sqrt{\pi}}{3}\sqrt{\frac{2R_0^6 t}{\tau}}\left(\frac{3}{2}\right)^{1/2}\langle\kappa^2\rangle^{1/2}} \tag{S9}$$

Now, let's define two new parameters, c and γ_d:

$$c = \frac{4\pi}{3}\rho R_0^3 \tag{S10}$$

$$\gamma_d = \sqrt{\frac{\pi}{2\tau}}c\left(\frac{3}{2}\right)^{1/2}\langle\kappa^2\rangle^{1/2} \tag{S11}$$

The dimensionless parameter c refers to the number of fluorophores within the 3-dimensional sphere volume of radius R_0.

Since we are describing a 3-dimensional isotropic system in the dynamic averaging regime, $\langle\kappa^2\rangle$ is replaced by 2/3(5–7). Thus γ_d turns into $\gamma_d = \sqrt{\frac{\pi}{2\tau}}c$. Introducing equations (S10 and S11) in equation S9, the emission anisotropy is given as follows(1, 2, 8):

$$r(t) = r_0 e^{-\gamma_d t^{1/2}} \tag{S12}$$

If we refer to the exponent of t in equation S12 as δ, we can see that for a 3-dimensional spatial distribution given by $u(R) = 4\pi R^2$, δ=1/2. If this distribution was assessed in a 2-dimensional scenario ($u(R) = 2\pi R$), δ would be equal to 1/3(1, 5).

In the previous analysis, the orientation factor κ^2 was averaged to 2/3 due to the rapid rotation of the fluorophore in comparison to its FRET rate constant. The system was said to be in the so-called dynamic averaging regime. However, when the fluorophores are strictly frozen during their fluorescence lifetime ($\tau \ll \theta$), the system is said to be in the static averaging regime. In this case, the orientation factor $\kappa^2(\Omega)$ has to be averaged over the angular distribution given by $\nu(\Omega)$ as follows(5):

$$lnH(t) \approx -\frac{\rho}{2}\int_0^{\infty}\int_{\Omega}\Big(1-e^{-2\frac{3}{2}\frac{1}{\tau}\left(\frac{R_0}{R}\right)^6\kappa^2(\Omega)t}\Big)u(R)dR\nu(\Omega)d\Omega \tag{S13}$$

Applying the same method as the applied for the dynamic averaging system, the fluorescence anisotropy takes the following form:

$$r(t) = r_0 e^{-\sqrt{\frac{\pi}{2}}c\left(\frac{3}{2}\right)^{1/2}\langle|\kappa|\rangle(\frac{t}{\tau})^{1/2}} = r_0 e^{-\gamma_{st}t^{1/2}} \tag{S14}$$

where:

$$\langle|\kappa|\rangle = \int_{\Omega}[\kappa^2(\Omega)]^{1/2}\nu(\Omega)d\Omega \tag{S15}$$

and the new γ is:

$$\gamma_{st} = \sqrt{\frac{\pi}{2\tau}}c\left(\frac{3}{2}\right)^{1/2}\langle|\kappa|\rangle \tag{S16}$$

Therefore, we see that the parameter γ in the dynamic and static averaging regimes differs in a scaling factor μ:

$$\mu = \frac{\gamma_{st}}{\gamma_d} = \frac{\langle|\kappa|\rangle}{\langle\kappa^2\rangle^{1/2}} \tag{S17}$$

Thus, the fluorescence anisotropy of the system in the dynamic regime is dictated via γ by the orientation factor $\langle\kappa^2\rangle$, while $\langle|\kappa|\rangle$ dictates the fluorescence anisotropy decay of a static averaging system. We know that for a dynamic averaging 3-dimensional system, $\langle\kappa^2\rangle$ can be reduced to 2/3. In the static limit, when the system is isotropic and 3-dimensional, and the orientation factor describes a 3-dimensional distribution, $\langle|\kappa|\rangle$ can be calculated, yielding $\langle|\kappa|\rangle = 0.6901$. This is the same as saying that $\langle\sqrt{\kappa^2}\rangle^2 = 0.6901^2 = 0.4762$, with $\mu = 0.8452$. If the space is the same but the orientation factor describes a 2-dimensional distribution, then $\langle|\kappa|\rangle = 0.7397$, where $\langle\sqrt[3]{\kappa^2}\rangle^3 = 0.7397^3 = 0.4047$ and with $\mu = 0.8468$(5, 6).

An identical method to calculate the FRET efficiency of the entire system in either of the two regimes, dynamic or static, is now described. Let's define the fluorescence intensity decay of the contribution of all initially excited fluorophores (donors) in a system as follows:

$$I(t) = I_0 e^{-t/\tau}H(t) \tag{S18}$$

where I_0 is the fluorescence intensity at $t = 0$ and τ is the fluorescence lifetime of the fluorophore. Because $H(t)$ does not take into account the finite fluorescence lifetime τ, an additional exponential term with the fluorescence lifetime is added in the equation. Since the initial excited fluorophores contribute predominantly to the fluorescence anisotropy, a relationship between their fluorescence steady-state anisotropy r and quantum yield Φ can be established(2):

$$\frac{r}{r_0} = \frac{\Phi}{\Phi_T} \tag{S19}$$

where r_0 is the fluorescence anisotropy of the system in the absence of FRET and Φ_T is the total quantum yield.

If we consider the fluorescence intensity of the entire system $I_T(t)$ to decay as a single exponential function (an average fluorescence lifetime may be also introduced as an approximation), equation S19 takes the form(2, 9):

$$\frac{r}{r_0} = \frac{\Phi}{\Phi_T} = \frac{\int_0^{\infty}dtI(t)}{\int_0^{\infty}dtI_T(t)} = \frac{1}{\tau I_0}\int_0^{\infty}dtI(t) = \frac{1}{\tau}\int_0^{\infty}dte^{-t/\tau}H(t) \tag{S20}$$

Introducing the explicit expression of the probability $H(t)$ within equation S20, the result is(2):

$$\frac{r}{r_0} = \frac{1}{\tau}\int_0^{\infty}dte^{-t/\tau}H(t) = 1 - f(y) \tag{S21}$$

where

$$f(y) = \sqrt{\pi}ye^{y^2}[1 - erf(y)] \tag{S22}$$

$$erf(y) = \frac{2}{\sqrt{\pi}}\int_0^{y}dte^{-t^2} \tag{S23}$$

$$y = \frac{\sqrt{\tau}}{2}\gamma \tag{S24}$$

The FRET efficiency of the donor fluorophores will be given by $f(y)$:

$$E_{FRET} = f(y) = \sqrt{\pi}ye^{y^2}[1 - erf(y)] \tag{S25}$$

This expression (equation S25) is equivalent to $E_{FRET} = 1 - \Phi$, where the quantum yield of the entire system is $\Phi_T = 1$.

2. Linker choice and secondary structure

The details of the linker for the simulations were chosen on the basis of the real linker in the experimental work. A (GGGGS)3 is a fairly standard flexible linker for joining two domains to allow them to behave independently.(10) The construct used is similar to constructs previously made and used for hetero-FRET studies.(11) If the linker is longer, the distance between the two GFPs, on average, increases which leads to a lower FRET efficiency.

The secondary structure propensity for linker residues in the GFP dimer, expressed as simulation fractions is shown in Table S6. These propensities are calculated according to the DSSP method of Kabsch and Sander(12). We can clearly see that the linker's secondary structure is dominated by turns and bends, as expected. The effect of this on the FRET efficiency will be via the orientation factor κ^2, as shown in Figure 10 of the main text.

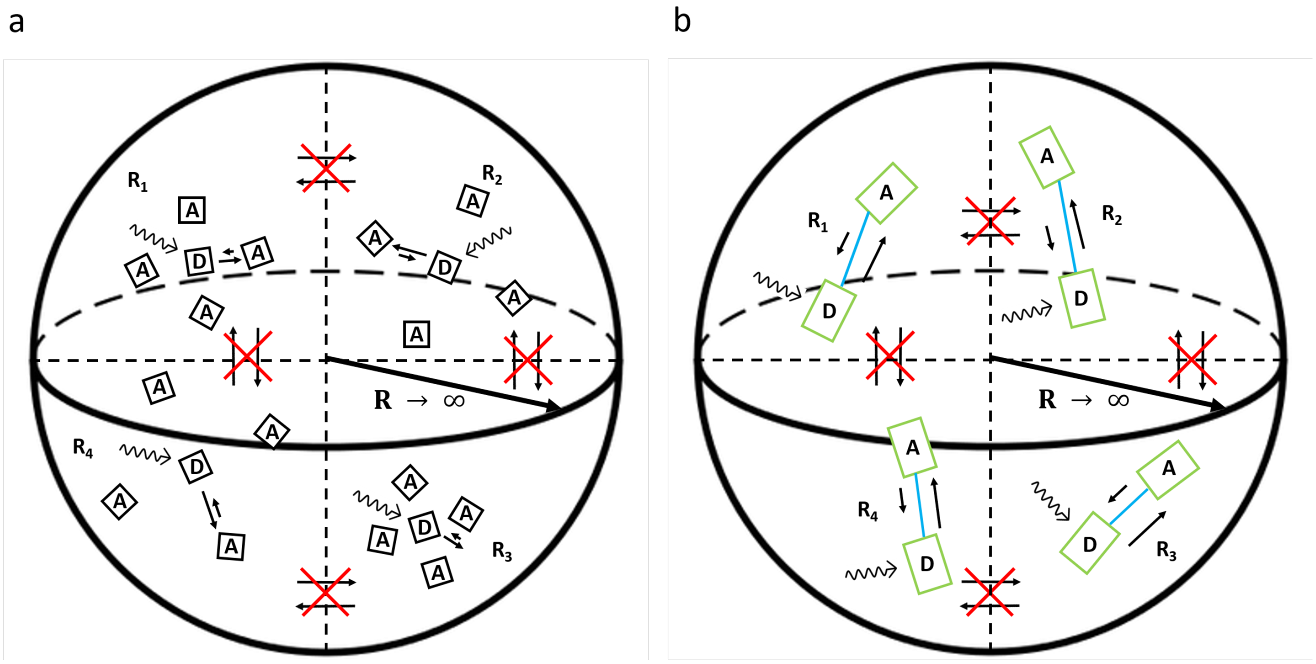

Figure S1: (a) Simplified graphical representation of the model described by Huber-Hamilton-Barnett(3) to quantify homo-FRET in a 3-dimensional diluted solution between identical fluorophores. The volume is divided in different regions determined by local concentrations and thus distances between fluorophores (R_1, R_2, R_3 and R_4). In each region a donor fluorophore is located in the middle and surrounded by acceptor fluorophores differently oriented. At time t a single interaction between donor and nearby acceptor takes place. The large arrow between donor and acceptor describes the transfer of energy via FRET from donor to acceptor. The short arrow represents the donor's re-excitation due to the transfer of energy via FRET from acceptor to donor. The pairs of arrows linking regions and crossed out in red represents the non-possibility of transferring energy via FRET between fluorophores of different regions. (b) Simplified graphical representation of the sample studied in this work, formed by a diluted solution of eGFP15eGFPs (two monomeric eGFPs (green) tethered by a linker of 15 aminoacids (blue)). This system corresponds to an analogy to (a), with the only difference that only two fluorophores (donor and acceptor at time t) are encountered for each region.

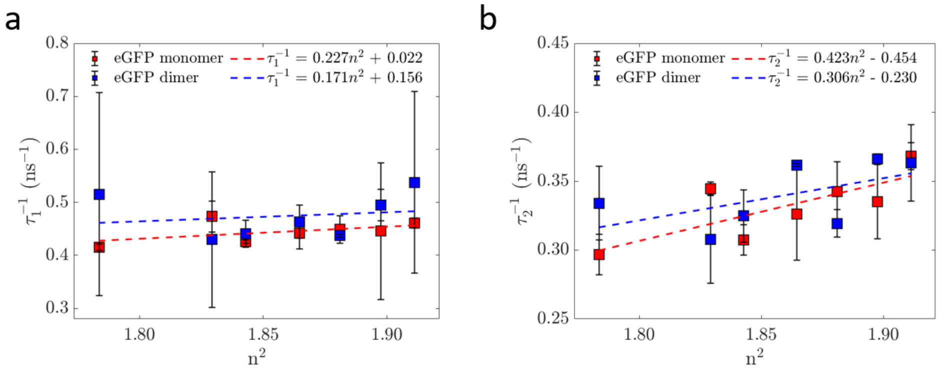

Figure S2: The short and long fluorescence lifetime of the GFP monomer and dimer from a double exponential fit to their fluorescence decays plotted versus the square of the refractive index, n^2. (a) Inverse of the shorter fluorescence lifetime plotted against the square of the refractive index. (b) Inverse of the longer fluorescence lifetime plotted against the square of the refractive index.

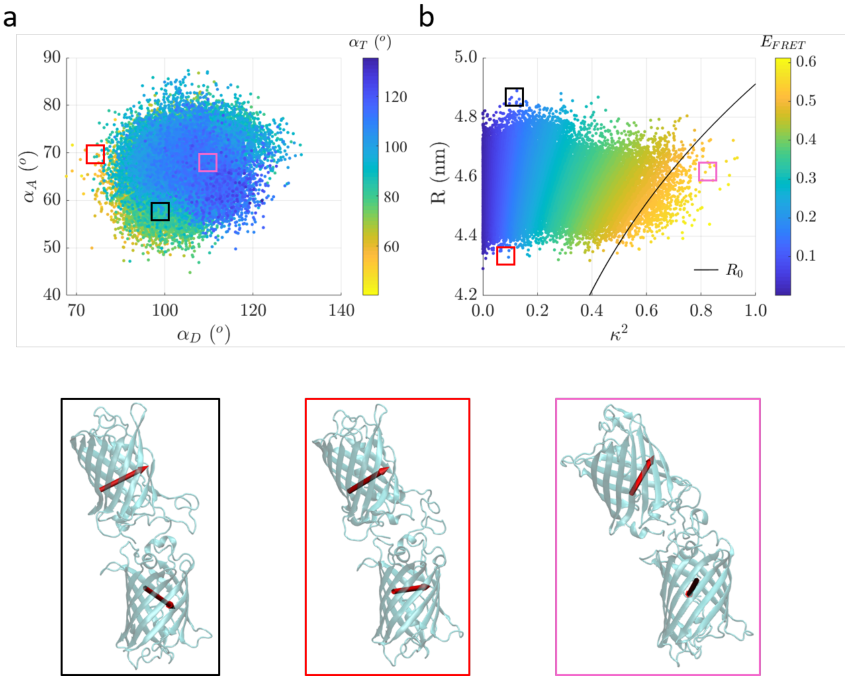

Figure S3: Scatter plots of (a) α_D, α_A and α_T, and (b) κ^2, fluorophore separation R and E_{FRET} of the GFP15GFP dimer. The GFP Förster distance R_0 as a function of κ^2 according to equation 7 is indicated by a line which follows the orange $E_{FRET} = 0.5$ shading. (c) Representative orientations of the two proteins from the differently coloured squares in the scatter plots in (a) and (b). The red arrows correspond to the GFP transition dipole moment $\vec{\mu}$, which indicates the fluorophore orientation of the protein.

Table S1: Fit parameters extracted from the fluorescence decays of a eGFP monomer and dimer (eGFP15eGFP) in solution. A double exponential decay model was used to fit the decays, measured with a single detector, according to equation 15 and the average fluorescence lifetime was calculated using equation 16. The average fluorescence lifetime and its reciprocal for the eGFP monomer and dimer are given by τ_{av} and τ_{av}^{-1}. n is the refractive index of the solution, and χ_R^2 is the goodness of the fit. The results are graphically shown in Figure 4c.

			**eGFP monomer**			**eGFP dimer**		
**% glycerol**	**n ± 0.001**	**n^2 ± 0.001**	τ_{av} (ns)	χ_R^2	τ_{av}^{-1} (ns^{-1})	τ_{av} (ns)	χ_R^2	τ_{av}^{-1} (ns^{-1})
0	1.336	1.784	2.59 ± 0.02	1.34	0.386 ± 0.003	2.52 ± 0.06	1.42	0.396 ± 0.010
5	1.353	1.829	2.54 ± 0.01	1.34	0.394 ± 0.001	2.52 ± 0.01	1.26	0.398 ± 0.001
10	1.358	1.843	2.53 ± 0.01	1.30	0.395 ± 0.001	2.50 ± 0.01	1.42	0.401 ± 0.001
15	1.366	1.865	2.52 ± 0.02	1.29	0.396 ± 0.002	2.49 ± 0.02	1.08	0.402 ± 0.003
20	1.372	1.881	2.52 ± 0.01	1.25	0.398 ± 0.001	2.45 ± 0.02	1.28	0.408 ± 0.003
25	1.378	1.898	2.50 ± 0.01	1.26	0.401 ± 0.001	2.45 ± 0.01	1.17	0.409 ± 0.002
30	1.383	1.911	2.46 ± 0.01	1.17	0.407 ± 0.002	2.38 ± 0.04	1.19	0.420 ± 0.007

$\Delta n^2 = \sqrt{2n}\,\Delta n$

Presented errors are associated to standard deviations of average values

Table S2: Fit parameters derived from time-resolved fluorescence anisotropy measurements of a eGFP monomer in solution. A representative time-resolved fluorescence anisotropy decay along with the fit model are shown in Figure 6a. The sample composition is given by its percentage of glycerol and viscosity η. The fit parameter r_0 corresponds to the initial anisotropy, θ is the rotational correlation time and the goodness of the fit is given by χ_R^2. The relationship between the rotational correlation time θ and the solution viscosity η is depicted in Figure 6d in green square data points and a dashed black line, with a fit model whose gradient is 18.1 ns/cP.

**% glycerol**	η **(cP)**	r_0	θ **(ns)**	χ_R^2
0	0.98	0.322 ± 0.002	16.5 ± 0.2	1.15
5	1.06	0.337 ± 0.001	21.4 ± 0.4	1.18
10	1.25	0.331 ± 0.000	23.4 ± 0.3	1.20
15	1.48	0.335 ± 0.010	27.6 ± 2.0	1.17
20	1.78	0.334 ± 0.001	38.6 ± 1.3	1.11
25	2.15	0.342 ± 0.001	41.9 ± 1.5	1.13
30	2.65	0.343 ± 0.000	47.4 ± 0.6	1.17
35	3.30	0.327 ± 0.003	67.9 ± 2.0	1.12
45	5.41	0.346 ± 0.002	97.6 ± 5.6	1.13
50	7.13	0.342 ± 0.001	123 ± 6	1.19

Table S3: Fluorescence parameters extracted from the fit of time-resolved anisotropy measurements of the eGFP dimer in solution using a double exponential and a stretched exponential decay model. The fit parameters for the double exponential decay model (equation 13) are: rotational correlation time θ, the inverse FRET rate constant ϕ and initial anisotropy values associated with each exponential, r_{01} and r_{02}. Figure 6d shows the dependence of the eGFP dimer rotational correlation time θ with the solution viscosity η (red square data points). The dependence of the inverse FRET rate constant ϕ on the viscosity η is shown in Figure 6e. For the stretched exponential decay model (equation 10), the fit parameters are: the initial anisotropy r_0 and the static γ_{st} parameter found within the exponential. The dimensionality parameter δ was fixed at 0.5. The dimensionless parameter c was calculated from γ_{st} with $\langle|\kappa|\rangle = 0.69$, and the goodness of both fits is indicated by χ_R^2. The dependence of γ_{st} and c on viscosity η is shown in Figure 6f.

	**Double exponential**					**Stretched exponential**			
**% glycerol**	r_{01}	θ **(ns)**	r_{02}	ϕ **(ns)**	χ_R^2	r_0	γ_{st}	χ_R^2	c
0	0.254 ± 0.007	17.2 ± 1.1	0.047 ± 0.003	0.51 ± 0.05	1.15	0.311 ± 0.002	0.229 ± 0.012	1.17	0.351 ± 0.012
5	0.249 ± 0.004	22.1 ± 1.2	0.054 ± 0.000	0.77 ± 0.11	1.20	0.315 ± 0.005	0.211 ± 0.005	1.21	0.323 ± 0.006
10	0.254 ± 0.004	23.9 ± 1.6	0.054 ± 0.006	0.78 ± 0.23	1.20	0.316 ± 0.004	0.198 ± 0.008	1.22	0.301 ± 0.010
15	0.245 ± 0.002	26.4 ± 1.3	0.050 ± 0.006	0.74 ± 0.11	1.20	0.302 ± 0.003	0.185 ± 0.006	1.21	0.279 ± 0.008
20	0.253 ± 0.003	28.4 ± 2.1	0.053 ± 0.005	0.70 ± 0.13	1.15	0.310 ± 0.003	0.177 ± 0.007	1.17	0.265 ± 0.009
25	0.263 ± 0.001	31.3 ± 1.2	0.055 ± 0.004	0.76 ± 0.09	1.16	0.321 ± 0.002	0.168 ± 0.003	1.17	0.251 ± 0.004
30	0.269 ± 0.004	33.1 ± 3.0	0.045 ± 0.003	0.68 ± 0.17	1.12	0.317 ± 0.002	0.147 ± 0.003	1.14	0.218 ± 0.004
35	0.264 ± 0.001	37.9 ± 0.2	0.045 ± 0.002	0.78 ± 0.04	1.16	0.313 ± 0.004	0.140 ± 0.004	1.16	0.207 ± 0.006
45	0.267 ± 0.001	44.0 ± 2.4	0.044 ± 0.004	0.72 ± 0.04	1.14	0.313 ± 0.002	0.125 ± 0.004	1.18	0.181 ± 0.006
50	0.266 ± 0.000	48.2 ± 2.0	0.042 ± 0.003	0.75 ± 0.15	1.13	0.310 ± 0.002	0.120 ± 0.004	1.18	0.181 ± 0.005

Table S4: The dimensionality parameter δ from the stretched exponential decay model (equation 10) when allowed to float freely in the fit of the GFP dimer anisotropy decays. This data is plotted in Fig 7.

**% glycerol**	η **(cP)**	δ
0	0.98	0.71 ± 0.12
5	1.06	0.58 ± 0.01
10	1.25	0.60 ± 0.01
15	1.48	0.34 ± 0.04
20	1.78	0.50 ± 0.10
25	2.15	0.60 ± 0.07
30	2.65	0.70 ± 0.07
35	3.30	0.88 ± 0.18
45	5.41	0.59 ± 0.02
50	7.13	0.69 ± 0.12

Table S5: FRET energy efficiency E_{FRET} of the eGFP dimer in solution and calculated from the fit parameters derived from time-resolved fluorescence anisotropy measurements. The double exponential decay model and stretched exponential decay models were applied. The fluorescence decays of the monomeric GFP were created from the parallel and perpendicular decays according to the denominator in equation 8. Equation 14 was applied to calculate the FRET efficiency when the anisotropy data was fitted with a double exponential decay model, equation 13. For the stretched exponential decay model, E_{FRET} was calculated according to equation 12. The fluorescence lifetime of the eGFP monomer in the absence of the acceptor is given by τ (equation 16). The results are presented graphically in Figure 8.

				**Double exponential**	**Stretched exponential**
**% glycerol**	**η (cP)**	**n ± 0.001**	**τ (ns)**	E_{FRET}	E_{FRET}
0	0.98	1.336	2.65 ± 0.01	0.723 ± 0.001	0.316 ± 0.010
5	1.06	1.353	2.63 ± 0.00	0.631 ± 0.000	0.303 ± 0.005
10	1.25	1.358	2.60 ± 0.00	0.624 ± 0.000	0.286 ± 0.009
15	1.48	1.366	2.56 ± 0.04	0.634 ± 0.003	0.269 ± 0.008
20	1.78	1.372	2.52 ± 0.02	0.644 ± 0.002	0.259 ± 0.009
25	2.15	1.378	2.51 ± 0.03	0.624 ± 0.003	0.247 ± 0.004
30	2.65	1.383	2.46 ± 0.02	0.645 ± 0.002	0.219 ± 0.004
35	3.30	1.389	2.47 ± 0.01	0.612 ± 0.001	0.210 ± 0.006
45	5.41	1.403	2.39 ± 0.01	0.625 ± 0.001	0.187 ± 0.006
50	7.13	1.407	2.41 ± 0.02	0.617 ± 0.002	0.184 ± 0.006

No error associated with viscosity η
Presented errors are associated with standard deviations of average values

Table S6: Secondary structure propensity for linker residues in the GFP dimer, expressed as simulation fractions.

Residue	Parallel β-sheet	Anti-parallel β-sheet	3-10 helix	α-helix	π (3-14) helix	Turn	Bend
GLY229	0.0008	0.0005	0.0544	0.0006	0.0004	0.0707	0.0000
GLY230	0.0238	0.0023	0.0674	0.0012	0.0008	0.2864	0.2109
GLY231	0.0019	0.0075	0.1034	0.0058	0.0008	0.3110	0.2614
GLY232	0.0068	0.0130	0.0695	0.0350	0.0008	0.2348	0.4227
SER233	0.0002	0.0051	0.1179	0.0391	0.0008	0.2940	0.3387
GLY234	0.0244	0.0102	0.0831	0.0393	0.0004	0.3727	0.2103
GLY235	0.0013	0.0143	0.0700	0.0369	0.0000	0.3527	0.1477
GLY236	0.0006	0.0034	0.0193	0.0347	0.0001	0.1594	0.4433
GLY237	0.0006	0.0098	0.0274	0.0256	0.0001	0.0950	0.5346
SER238	0.0005	0.0102	0.0174	0.0124	0.0001	0.0648	0.5049
GLY239	0.0031	0.0111	0.0188	0.0014	0.0001	0.1185	0.3628
GLY240	0.0030	0.0716	0.0260	0.0000	0.0001	0.1556	0.5908
GLY241	0.0001	0.0029	0.0259	0.0000	0.0000	0.6135	0.1715
GLY242	0.0035	0.0010	0.0178	0.0000	0.0000	0.5680	0.0000
SER243	0.0007	0.0668	0.0000	0.0000	0.0000	0.0000	0.0000

REFERENCES

1. Novikov, E. G., A. van Hoek, A. J. W. G. Visser, and J. W. Hofstraat, 1999. Linear algorithms for stretched exponential decay analysis. *Optics Communications* 166:189–198. https://doi.org/10.1016/S0030-4018(99)00262-X.

2. Bodunov, E. N., and M. N. Berberan-Santos, 2015. Stretched exponential kinetics of the luminescence concentration depolarization and penetration depth of molecules in a medium. *Optics and Spectroscopy (English translation of Optika i Spektroskopiya)* 119:22–28. https://doi.org/10.1134/S0030400X1507005X.

3. Huber, D. L., D. S. Hamilton, and B. Barnett, 1977. Time-dependent effects in fluorescent line narrowing. *Physical Review B* 16:4642–4650. https://doi.org/10.1103/PhysRevB.16.4642.

4. Lakowicz, J. R., 2006. Principles of fluorescence spectroscopy, 3rd edition. Springer, New York.

5. Baumann, J., and M. D. Fayer, 1986. Excitation transfer in disordered two-dimensional and anisotropic three-dimensional systems: effects of spatial geometry on time-resolved observables. *The Journal of Chemical Physics* 85:4087–4107. https://doi.org/10.1063/1.450880.

6. van der Meer, B. W., D. M. van der Meer, and S. S. Vogel, 2013. Optimizing the orientation factor kappa-squared for more accurate FRET measurements, in: *FRET - Förster Resonance Energy Transfer*, eds I. Medintz and N. Hildebrandt, John Wiley & Sons, Ltd, chapter 4, 63–104. https://doi.org/10.1002/9783527656028.ch04.

7. van der Meer, B. W., 2020. Kappaphobia is the elephant in the fret room. *Methods and Applications in Fluorescence* 8:030401. https://doi.org/10.1088%2F2050-6120%2Fab8f87.

8. Peterson, K. A., M. B. Zimmt, S. Linse, R. P. Domingue, and M. D. Fayer, 1987. Quantitative determination of the radius of gyration of poly(methyl methacrylate) in the amorphous solid state by time-resolved fluorescence depolarization measurements of excitation transport. *Macromolecules* 20:168–175. https://doi.org/10.1021/ma00167a028.

9. Förster, T., 1959. 10th Spiers Memorial Lecture. Transfer mechanisms of electronic excitation. *Discuss. Faraday Soc.* 27:7–17. https://doi.org/10.1039/DF9592700007.

10. Chen, X., J. L. Zaro, and W.-C. Shen, 2013. Fusion protein linkers: property, design and functionality. *Advanced Drug Delivery Reviews* 65:1357–1369. https://pubmed.ncbi.nlm.nih.gov/23026637.

11. Devauges, V., D. R. Matthews, J. Aluko, J. Nedbal, J. A. Levitt, S. P. Poland, O. Coban, G. Weitsman, J. Monypenny, T. Ng, and S. M. Ameer-Beg. Steady-state acceptor fluorescence anisotropy imaging under evanescent excitation for visualisation of FRET at the plasma membrane. *PLoS ONE* 9:e110695. https://doi.org/10.1371/journal.pone.0110695.

12. Kabsch, W., and C. Sander, 1983. Dictionary of protein secondary structure: Pattern recognition of hydrogen-bonded and geometrical features. *Biopolymers* 22:2577–2637. https://onlinelibrary.wiley.com/doi/abs/10.1002/bip.360221211.
